# Ecosystem services and justice of protected areas: the case of Circeo National Park, Italy

**DOI:** 10.1080/26395916.2021.1946155

**Published:** 2021-07-21

**Authors:** Stefania Benetti, Johannes Langemeyer

**Affiliations:** a Department of Methods and Models for Economy, Territory and Finance (MEMOTEF), University of Rome La Sapienza, Rome, Italy; b Department of Sociology and Social Research, University of Milano‐Bicocca, Milano, Italy; c Institute of Environmental Science and Technology (ICTA), Universitat Autònoma de Barcelona (UAB), Cerdanyola del Vallès, Spain; d Humboldt Universität zu Berlin, Institute of Geography, Berlin, Germany

**Keywords:** Matthias Schröter, Environmental conservation, social-ecological systems, social equity, natural resources management, shifting ecologic values, stakeholder perceptions, Aichi Target 11

## Abstract

Protected areas are key instruments for conserving biodiversity and landscapes. Yet, conservation initiatives are still often struggling to accommodate people’s needs, provoking conflicts, and lacking support from local communities. Our study combines environmental justice and ecosystem services approaches to provide a critical understanding of trade-offs between people’s interests and conservation goals in the case study of *Circeo National Park* (Italy). Applying a qualitative content analysis of different materials and using a survey of local residents, we focus on three main objectives: analysing the implementation of the ecosystem services framework in policy documents and exploring how different people value benefits from nature; investigating the decision-making process in terms of participation, information and communication strategies; and identifying how conservation policies generated different allocations of benefits, burdens and inequalities among social groups. The integrated approach applied in our study highlights ways to systematically uncover perceived injustices and identifies potential conflict lines. In the long run, this approach might help to increase the public acceptance of protected areas by fostering sustainability also in its often-overlooked social dimension.

## 1. Introduction

The designation of protected areas (PA) is one of the most important environmental conservation strategies globally (Palomo et al. 2013). PAs not only contribute to habitat provision for endangered wildlife but also climate change mitigation, carbon emission reductions and the generation of economic benefits, such as the promotion of tourism (Andam et al. 2010; Watson et al. 2014). The United Nation’s Convention on Biological Diversity (CBD) states that PAs must be managed effectively and equitably by 2020 (Aichi Target 11), framing PAs as well-connected and integrated systems within wider land and seascapes (CBD, 2010, p. 9). Furthermore, the Post-2020 Global Biodiversity Framework, currently in ongoing development, recommend safeguarding PAs in partnership with indigenous peoples, local communities, and other owners or managers, underlining the importance of protected sites for both biological and cultural diversity (CDB, 2020, p. 12).

Yet, a major criticism of PAs is the focus on geographically limited and static ecosystems (Petrosillo et al. 2009; Palomo et al. 2013; García-Llorente et al. 2018). For decades, environmental conservation followed the paradigm to safeguard biodiversity from human threats through the exclusion of people from PAs (Mcdonald et al. 2008; Martin et al. 2016), generating the perception of a conflicting vision of conservation versus development (Palomo et al. 2011). This nature-people dichotomy poorly reflects the reality of many PAs, where people are essential as stewards of biodiversity (Reed 2008; Andrade and Rhodes 2012; Palomo et al. 2015), and protected ecosystems are essential to people’s well-being and livelihoods through the provision of multiple ecosystem goods and services (Trzyna 2007; UNEP-WCMC and IUCN 2016). As a consequence, PAs often lack support by local communities (MacDonald et al. 2013; García-Llorente et al. 2018), and may be perceived as unjust in the face of people’s capacities to flourish in equal terms (Fabinyi et al. 2014; Gurney et al. 2014).

A better understanding of perceived (in)justice with regard to benefits and burdens of PAs is suggested as a key mechanism through which a more integrated social-ecological vision and collective action can be fostered (Zafra-Calvo et al. 2017). While the social-ecological interdependencies characterizing PAs are increasingly accounted for, a divergent understanding of how PAs impact different social groups and whether this is perceived as fair is still widely lacking (but see Dawson et al. 2017; Ward et al. 2018). As a consequence, local PAs managers often do not share the conservation objectives, and social acceptance for conservation remains low among local communities, which hampers the effectiveness of PAs or requires top-down enforcement. In the long run, conservation strategies are jeopardized, or at least rendered less efficient, without community support and trust (Hockings et al. 2006; Palomo et al. 2013).

Counteracting inefficient PAs requires an integration of ecological goals alongside social justice objectives across different spatial and temporal scales (*cf*. García-Llorente et al. 2018; Langemeyer and Connolly 2020). In this context, the spread of ecosystem services (ES) governance, accounting for the benefits people obtain from ecosystems and whether and how a PA affects them, may support effective and lasting conservation strategies (Sikor et al. 2014). ES approaches support broad and diverse accounting for multiple direct and indirect benefits of PAs for local people’s well-being, including a sense of place, opportunities to learn about nature, the mitigation of climate change, the protection of threatened species and habitats that have meaning for people, while supporting local economies through nature tourism (Trzyna 2014). ES assessments can support decision-making, policy design, and action with respect to natural resource management (Primmer et al. 2015; Sattler et al. 2018), by visualizing social-ecological dependencies across space and scales (Barnaud et al. 2018), and thereby, reducing the risk of narrow ecological objectives in the management of PAs (Palomo et al. 2013).

However, while ES management is on the rise, ES approaches tend to have a blind spot for social injustices and trade-offs among different groups of society (Chaudhary et al. 2018; Turkelboom et al. 2018), neglecting social diversity in demands and power structures determining access to ES benefits (Iniesta-Arandia et al. 2014; Chaudhary et al. 2018; Díaz et al. 2018; with the exception of literature on payments for ES, *e.g*. Sikor 2013). To overcome these limitations deeply rooted in ES scholarship (*e.g*. Gómez-Baggethun et al. 2010; Díaz et al. 2018; Langemeyer and Connolly 2020), we suggest a combination of ES and environmental justice (EJ) approaches (*cf*. Schlosberg 2001; Chaudhary et al. 2018). EJ thinking can extend ES assessments towards the analysis of the distribution of benefits and burdens to different people (*e.g*. Lele 2013; Sikor et al. 2014; Dawson et al. 2017), and supports the acknowledgement and incorporation of diverse interests in conservation strategies, in order to uncover and manage open and latent social conflicts (Kovács et al. 2015; Hanaček et al. 2021).

The main purpose of this article is to empirically investigate a case study in which the integration of EJ thinking into ES-based management was applied, with the aim of increasing social support for nature conservation through PAs. Following this general aim, we address the case study of *Circeo National Park* (CNP) in Italy under three specific objectives: (a) understanding the implementation of ES concepts and exploring ES socio-cultural values (recognition justice); (b) investigating decision-making procedures behind them in terms of participation and information and communication approaches (procedural justice); and (c) identifying how conservation policies generate social inequalities and spatial burden shifting (distributional justice).

## 2. Conceptual framework

**Figure 1.**
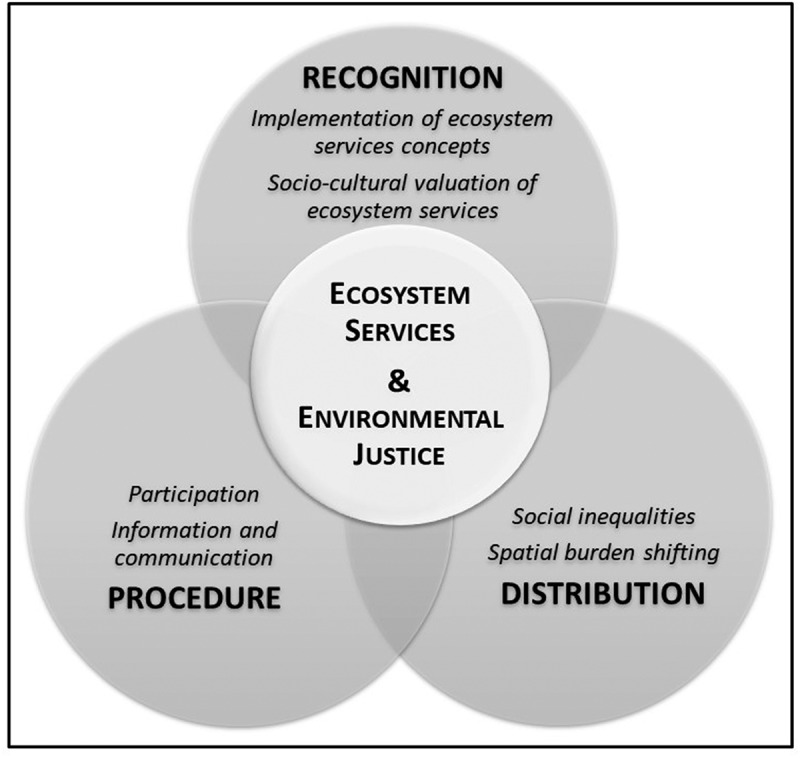
The integrated ecosystem service and environmental justice framework, adapted based on Langemeyer and Connolly (2020). The study addresses the three main pillars of environmental justice: recognition justice, procedural justice, and distributional justice

Social diversity in a community implies diversity in terms of those who benefit (or not) from ES, as well as in terms of what is considered fair, both for the distribution of ES and their governance (Lau et al. 2018). Building on established EJ scholarship, this study addresses the three main pillars of justice that have previously been related to ES (Sikor et al. 2014; Martin et al. 2015; Zafra-Calvo et al. 2017; Chaudhary et al. 2018): recognition justice, procedural justice, and distributional justice. The study aims to contribute to the growing research area of ‘ecosystem services justice’ (*e.g*. Sikor 2013; Baró et al. 2021; Calderón-Argelich et al. 2021) by proposing and operationalizing an integrated and empirical investigation to ES and EJ, as shown in the conceptual framework in Figure 1.

Within this framework, recognition justice refers to which stakeholders (and their objectives) are considered in ES management (Chaudhary et al. 2018), acknowledging people’s distinct identities (He and Sikor 2015) and differences in their understanding of ES benefits and needs, as well as preferences related to ES (Langemeyer and Connolly 2020). Diverse socio-cultural or ecological values preferences can often be a cause of conflict (Dawson et al. 2017; Hanaček and Rodríguez-Labajos 2018), as stakeholders have differing views on the environment. Furthermore, in PAs, understanding relational values is essential (Vos et al., 2018), expressing how people appreciate nature through their relations and interactions with it, by means such as place attachment, cultural identity and social cohesion (Arias-Arévalo et al. 2017; Chan et al. 2018; Himes and Muraca 2018). The lack of recognition of different perceptions of and interests in the benefits provided by a local ecosystem can lead to recognitional injustices (Sikor et al. 2014; Spangenberg 2015; Cáceres et al. 2015). Hence, identifying how different people value and prioritize ES is a crucial foundation for equitable ES management (Sikor et al. 2014).

Procedural justice instead refers to how decisions are made and enacted, including the framing of decision-making, transparent management and communication approaches, practices of conflict resolution, as well as the participation of stakeholders in decision-making processes (Gustavsson et al. 2014; Zafra-Calvo et al. 2017). Environmental management inevitably involves trade-offs among different objectives, values, and stakeholders (Daw et al. 2015), and decisions concerning ES are reflected in ecosystem governance and management tools. Therefore, procedural injustice may occur in cases with a lack of public participation in decision-making processes, vulnerable groups or different stakeholders are excluded from environmental management, or where there is ineffective communication and a lack of transparency and access to environmental information (Sikor 2013; Sikor et al. 2014; Aragão et al. 2016; Hanaček and Rodríguez-Labajos 2018).

Finally, distributional justice focuses on the allocation of benefits and burdens to different groups of a society (Schlosberg 2004; Boone et al. 2009; Martin et al. 2015; Chaudhary et al. 2018). It is a critical component of successful PAs management since unequal access to natural resources and ES constitutes a major cause for environmental conflicts (Hanaček and Rodríguez-Labajos 2018). Distributive fairness is related to the consideration of those who benefit from ES and who incur costs as a result of protecting the generation of ES (Aragão et al. 2016; Fleischer et al. 2018).

## 3. Methods

### 3.1. Study area

**Figure 2.**
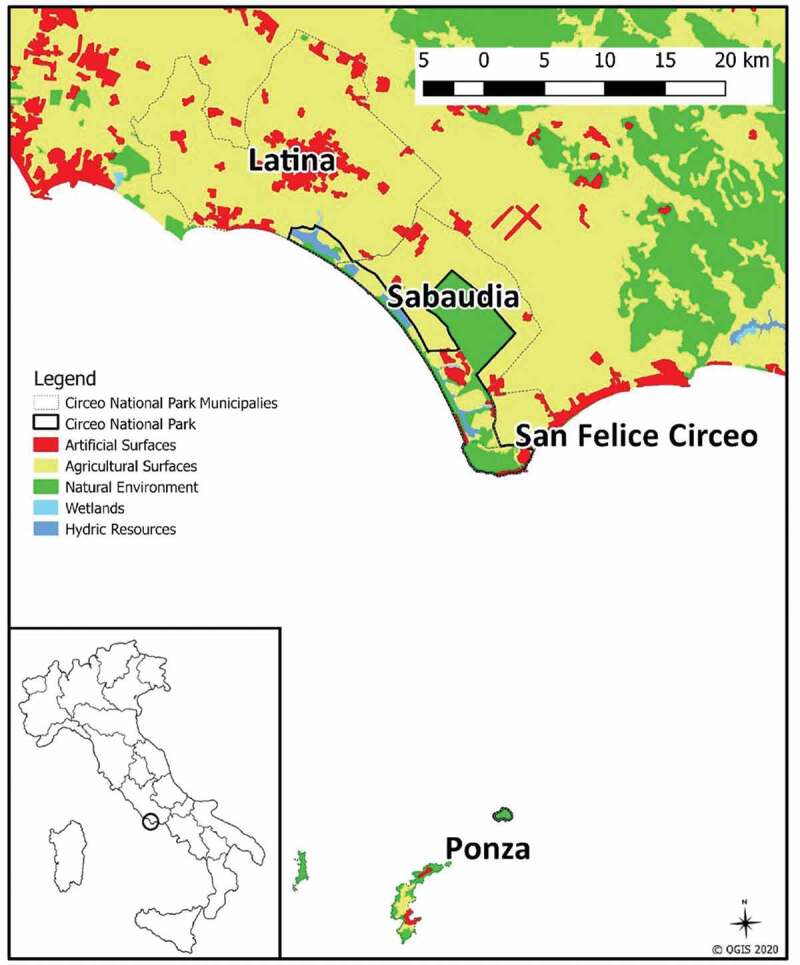
Location and land cover map of the Circeo National Park, Italy. Land cover information is based on the 1st hierarchical level of the Corine Land Cover legend (data source: MATTM, 2017; ISPRA, 2018)

The case study is focused on Circeo National Park (CNP), which is a typical example of nature conservation through the creation of a PA. The area covers 8,917 hectares and is located 100 km south of Rome on the Tyrrhenian Coast of Italy (Figure 2). In the past, the so-called Pontine Plain was characterized by a thick forest, marshland, and coastal lakes. This wild landscape remained unchanged until 1928 when drainage and clearing of the area began. In 1934, a small area of this plain forest was officially designated as protected under Italian National Law (L. 285/1934) in order to improve the flora and fauna, preserve the special geological formations and the beauty of the landscape, as well as to develop tourism.

Up until the establishment of a separate CNP Management Authority in 2005, the CNP was managed by the State Forestry Corps. In 2005, a new Park Authority was created (under Italian Law 394/1991, ‘Framework Law on Protected Areas’) for decision-making and the management of the National Park, replacing its previous governance structure. The Park Authority is composed of a President, the Governing Council, the Director, the Executive Council, and the Auditors Council, who are directly appointed and supervised by the government’s Minister of the Environment, in coordination with other national and regional institutions. The Park Authority also includes the Park Community, an advisory body composed of elected representatives, including the Presidents of the Regions, Presidents of the Provinces and Mayors of the municipalities intersecting the CNP territory. The principal tools of the Park Authority are the development of the CNP-Statute, the CNP-Regulation, the CNP-Plan, as well as the Long-term Economic and Social Plan, whereby the Park Authority guarantees citizens, associations, and collective subjects the right of requests, petitions, and proposals. The Park Authority and its instruments are described in further detail in Appendix I.

The CNP extends over four municipalities and is characterized by five different ecosystems: forest, coastal dunes, promontory, wetlands, coastal lakes, as well as the island of Zannone. In numerical terms, about 6.5% of the area of the CNP is characterized by artificial surfaces and urban areas (Garcia et al. 2013), while about 18% of the territory is allocated for agriculture activities (Ente Parco Nazionale del Circeo 2011a). Moreover, one of the lakes (Paola Lake) is private and, from 2007, the family owning it implemented a project for the environmental and productive restoration of the area, focused on some economic activities, such as aquaculture and tourism.

The total CNP population is 9.6% of the entire municipal population and is more concentrated in the municipality of Sabaudia and San Felice Circeo (see Appendix II for more details). For this reason, this study focuses on these two municipalities of the CNP. From an economic point of view, the main activities of the study area are agriculture, trade, and tourism, which are carried out in both municipalities, including some zones of CNP (*e.g*. the agricultural surfaces, highlighted in light yellow on Figure 2, are both inside the CNP boundaries and in the surrounding areas).

### 3.2. Data collection and analysis

The research applied a mixed-methods approach consisting of a qualitative content analysis of different materials – laws, policy documents, institutional websites, secondary data, grey literature and literature about the CNP case study – and a survey of 375 local residents, to address the three specific objectives of the study. First, and in order to analyse the recognition justice dimension, we used the content analysis to examine the implementation of the ES framework in various CNP policy documents to identify the consideration of ‘human well-being’, ‘values’, and ‘beneficiaries’; we complemented this information with insights from the survey on the local residents’ awareness and appreciation of ES. Second, the content analysis served to understand the decision-making processes regarding the CNP, who participated, and which information and communication approaches were adopted; while the survey was used to contrast with the citizens’ perceptions of these processes, in combination exploring the procedural justice dimension. Finally, the content analysis, as well as the survey information, was used to examine the distributional justice of ES and the allocation of benefits and burdens among different stakeholder groups.

#### 3.2.1. Qualitative content analysis

We used a qualitative content approach (*cf*. Bryman 2012, p. 557–559, 714) for the analysis of different materials (Appendix III summarizes the references used in this step). In particular, we selected the most recent official Italian laws about PAs and the CNP, policy documents available on the CNP institutional website, and the literature about the case study (excluding biological and zoological researches). The criterion of the most recent document has been applied also for secondary data about the study area obtained from the Ministry of the Environment and Protection of the Territory and the Sea, the National Institute of Statistics and the Higher Institute for Environmental Protection and Research websites, and from some personal communications. Furthermore, we used the CNP institutional website to read the online grey literature available for the period January 2016 – December 2018. Before focusing on the justice spheres, an initial screening of these materials was conducted in order to support a baseline understanding of the CNP.

First, to address the recognition justice dimension, our analysis investigated the level of implementation of the concepts related to ES within the core policy instruments of the CNP, while the longitudinal analysis of policy documents allowed examining the past recognition of ES *(cf*. Wilkinson et al. 2013). We selected documents dealing with park strategies particularly important to the society and economy of the study area: the CNP-Plan and the Environmental Strategic Assessment (Parco Nazionale Del Circeo 2016, 2017). We used the MAXQDA computer program to code the content of 16 documents (nine belonged to the CNP-Plan and seven to the Environmental Strategic Assessment) and we counted the frequencies of the terms ‘ecosystem services’, ‘benefits’, ‘human well-being’. Moreover, we codified and quantified the words associated with the concept of ‘values’ (for instance: naturalistic, historical, intrinsic values, etc.) and we differentiated these words into two typologies: those related to the environmental sphere and those related to the socio-economic sphere. Then, we systematically analysed the documents with the purpose to explore which potential beneficiaries of ES and which values of different social groups have been recognised by the Park Authority. We systematically searched for specific keywords (N≃40) to identify potential beneficiaries of ES (for instance, general categories, such as ‘local populations’, ‘civil society’, ‘associations’, ‘economic operators’, or specific social groups, such as ‘farmers’, ‘fishermen’, ‘young people’, ‘women’, etc.). Once we identified the sentences which referred to potential beneficiaries, we verified if these sentences were related to specific values (for instance, the ‘economic value of the CNP for farmers’). The qualitative content analysis further examined to which extent the CNP documents considered the potential negative effects on people’s livelihood implied by the conservation regime of the CNP. Finally, we coded the policies’ objectives into ES categories (Appendix IV), comparing them with international ES classifications (Common International Classification of Ecosystem Services – CICES, Haines-Young and Potschin 2011; The Economics of Ecosystems and Biodiversity – ; TEEB 2010). This exercise supported the definition of ES that would enter the valuation survey in the consecutive methodological step.

Addressing the procedural sphere of ES justice requires the involvement of different stakeholders and an examination of the social-political context, institutions, governance structures, and power relations of the decision-making processes (*cf*. Langemeyer and Connolly 2020). Therefore, we revised Italian laws on National Parks and all policy documents related to the CNP – including the regulation and the CNP-Plan – to compile background information on the past and current design of decision-making processes and the incorporation of different stakeholder groups and their interests. We identified institutions, regulations, and tools for the management of PA, and we verified their implementation in the CNP, in particular on the CNP-Plan.

Finally, the analysis related to the distributional justice aimed at identifying the existence of conflicts concerning the delivery and the conservation of different ES, as well as trade-offs between different societal groups regarding the benefits and burdens related to the protection measures of the CNP. First, we examined the content of the park policy documentations (Strategic Environmental Assessment and the CNP-Plan), identifying whether the conservation goals affected some specific social groups. Next, we deepened the information by selecting the pertinent grey literature available online (news and press review on the CNP website, Parco Nazionale del Circeo 2018a, 2018b), and by searching for keywords (N≃20) like ‘erosion of the dune’ or ‘agriculture’ and ‘wild boars’. Lastly, we integrated the contents with complementary information from scientific publications about the case study.

#### 3.2.2. Survey

The second step of our methodological approach consisted of designing and conducting a survey to local stakeholders and users of the CNP. Survey research is particularly useful for eliciting people’s attitudes and opinions about social, political, and environmental issues and is a tool for gathering information about people’s lives that is not available from published sources (McLafferty 2010). The survey was conducted in the form of questionnaires, that were pre-tested (N = 20) and distributed online, as well as in a paper version between December 18th, 2018 and February 18th, 2019. The survey URL link was published on the CNP website, and on Facebook profiles and community bulletin boards. Furthermore, it was sent by private messages to Facebook contacts and pages of associations, commercial and touristic activities. It is important to note that Facebook is more widely used in Italy compared to other European countries. In order to reach people who do not use the internet, which we assumed to be primarily related to advanced age, the paper version of the questionnaire was distributed in three elderly centres within the study area.^1^ Overall, 375 anonymous responses were collected, of which 52 were obtained from the elderly centres and 323 online. The composition of the sample population, restricted to individuals older than 18 years, is reported in Appendix V. Incomplete questionnaires (N = 10) were excluded from the analysis, resulting in 365 questionnaires that have been considered in this study. The questionnaire combined 15 multiple choice questions, nine multiple choice questions with multiple answers, and seven Likert scale questions, which generated answers that could be coded and processed quickly, and two open-ended questions, which aimed to capture the interviewee’s point of view through detailed and qualitative answers (McLafferty 2010; Bryman 2012). The entire questionnaire is available in Appendix VI.

The survey addressed the three dimensions of EJ, starting with recognition justice, in particular, related to the disaggregated recognition of ES values for different societal groups (Langemeyer and Connolly 2020). Before exploring respondents’ values about ES, the questionnaire included a set of multiple-choice questions regarding awareness of the benefits provided by the CNP, to evaluate respondents’ familiarity with the local context. For instance, in order to define the awareness of ES environmental education and science, the questionnaire asked ‘Do you consider the statement “some areas of Circeo National Park have educational and scientific value” is true?’ and had the possible answers, ‘yes’, ‘no’, and ‘I don’t know’. The appreciation of ES by citizens was assessed by means of a socio-cultural valuation approach that used Likert-scale rankings (*e.g*. Martín-López et al. 2012; Oteros-Rozas et al. 2014; Camps-Calvet et al. 2016). The socio-cultural assessment allowed us to explore respondents’ values regarding ES and to identify social groups that differently evaluated ES. Since people were not assumed to be familiar with the term ‘ecosystem services’ (*cf*. Plieninger et al. 2013), respondents received the following brief explanation: *‘The objective of this section is to evaluate the importance of benefits provided by the Circeo National Park for societal and individual well-being […]’*. Pre-testing showed this explanation to be sufficient to introduce a rough understanding of the ES concept; the actual questionnaire thus did not refer explicitly to the term ‘ecosystem services’. Pre-testing also showed the need to simplify the technical language used for the description of ES. For this reason, the questionnaire presented four tables, using simple descriptions and presenting examples and pictures of groups and single ES, identified through the grey literature review. The questionnaire examined the socio-cultural values attached to ES and their importance at the individual level, asking respondents to score each ES according to its importance for their personal well-being (not important = 1, minor importance = 2, significant = 3, or strong importance = 4). The survey further included a section on the socio-demographic characteristics of the respondents, including gender, age, place of residency, sector of employment, and type of work of the respondents. The data analysis followed the methods applied in Oteros-Rozas et al. (2014). We used a minimum-maximum normalization to standardize data on a 0 to 1 scale (Willemen et al. 2010; Castro et al. 2015), and we calculated the mean values and the standard deviation for each ES. The Pearson chi-square test (Bewick et al. 2003; Mchugh 2013) was used to explore associations between values of groups and single ES and some characteristics of respondents (proximity of residency to the CNP, gender, age, and business sectors). For all the tests, the significance level was fixed at 0.05.

Secondly, the survey addressed the procedural dimension of justice in terms of participation and relation with the CNP management from the respondents’ perspective. Respondents were asked about their participation in the CNP-Plan design and eventually why they did not participate. To this end, we developed a multiple-choice approach, with multiple answers to questions regarding the participation in park initiatives and events and the reason for the eventual non-participation. Moreover, respondents were asked to evaluate the framing and communication from CNP management in advertising and promoting participatory initiatives using Likert-scale rankings, which were also used to determine the respondents’ level of trust in the management of the park and in the resolution of problems and tensions with citizens.

Thirdly, the survey addressed the distributional justice dimension to examine potential negative impacts on citizen groups due to the protection regime of the park. Using a multiple-choice question, we asked whether and how the CNP had impacted the respondents or their family. Then, we left an open-ended question to the respondent to give them the chance to explain the reasons for the previous answer. The open answers were analysed using an interpretative analytical approach (*cf*. Thematic analysis by Bryman 2012, p. 578–581, 717), in which different responses were reviewed and coded to identify the main topics suggested by respondents; for instance, we coded the answer ‘the presence of the CNP limits tourism’ as ‘limitations for production activities and economic development’, or the response ‘the management is too bureaucratic and inefficient’ both in the topics ‘inefficiency of Park Authority’ and ‘high bureaucracy for permissions’.

Two major limitations in the data collection were related to the online sampling method, as well as the sampling within elderly centres, which can be framed as selection bias resulting from the volunteer sampling approach (Baltar and Brunet 2012). Selection bias concerns the tendency of some individuals to respond to an invitation to participate in a survey, while others ignore it, leading to a systematic bias (Wright 2006). In an attempt to address this limitation and to make the sample more representative, we applied post-stratification (Little 1993). We stratified the universe population of the study area and the sample population based on the municipality of residence, gender, and age. We calculated weights based on the ratios between the universe population and the sample population and weighted the responses of the sample population accordingly.

## 4. Results

### 4.1. Recognition justice

#### 4.1.1. Implementation of ecosystem services concepts

**Figure 3.**
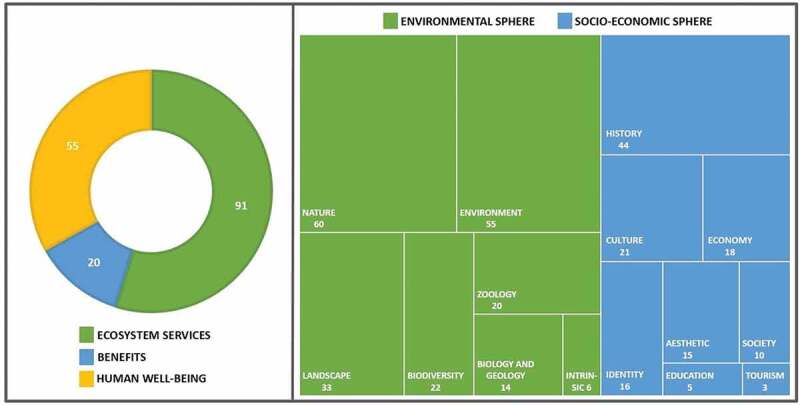
The word count in 16 documents of the Circeo National Park. On the left side, the frequencies of the terms ‘ecosystem services’, ‘benefits’, and ‘human well-being’. On the right side, the frequencies and the typologies of words associated with the concept of ‘values’

First, addressing the objective to explore the implementation of the ES context in CNP documents, our results reveal a fairly generic use of these concepts. In fact, the documents examined often referred to the conservation of ‘ecosystem services’ (N = 91 in Figure 3), guaranteed by biodiversity or by various ecosystems of the park, but without going into detail, specifying, for instance, the typology or the value. Likewise, the concept of ‘human well-being’ (N = 55) was not fully explored. The main reference was to the park’s goal of halting biodiversity loss and supporting ES for human well-being, without any additional detailed information. The term ‘benefits’ had fewer citations (N = 20), nevertheless, its use was less vague, with references to benefits in the management of tourist presences, benefits of future generations, and benefits, such as the production of energy from waste material. However, even in this case, quantitative references were lacking. The concept of ‘value’ was also used in a generic way, underlining, in particular, the natural (N = 60), environmental (N = 55), and historical (N = 44) value of the park, but without describing for whom this applied or quantifying it. What can be clearly seen in Figure 3 is the dominance of the environmental sphere associated with the idea of ‘value’ rather than the socio-economic field.

The qualitative content analysis revealed that potential beneficiaries of ES are partially accounted for in the CNP documents. We identified references to overall categories, such as ‘local populations’ for provisioning services, ‘populations of adjacent urban settlements’ for air purification and ‘future generations ‘ for cultural services. Explicit references were made to the recognition of economic benefits for certain categories, such as ‘farmers and breeders’, ‘tour operators’, ‘commercial operators of fishing tourism’, and ‘the owners of Paola Lake’ (*cf*. Ente Parco Nazionale del Circeo 2011b; Parco Nazionale Del Circeo 2016). However, the content analysis of the documents did not reveal whether the strategies adopted by the park generated burdens or disadvantages in relation to the various ES, neither for the community as a whole nor for some specific social groups.

In terms of specific ES, the CNP-Plan and the Strategic Environmental Assessment strictly referred to cultural ES as a specific conservation objective as vaguely defined benefits of future generations. For instance, one of the CNP-Plan goals concerns the preservation of the landscape, archaeological, historical and cultural assets, while the Strategic Environmental Assessment highlighted the importance of the CNP for different touristic activities, including ecotourism, environmental education, and research, thus focusing on benefits mainly provided to people distant to the CNP region. Other policies, for example, those addressing sustainable agriculture, agritourism activities, aquaculture, and sustainable mushroom harvest in the forest, were related to provisioning ES with strong cultural connotations for local communities, particularly agricultural food production, aquaculture, and mushroom picking. Except for air purification – mainly benefiting the population in adjacent urban settlements – the CNP policy strategies did not clearly consider the benefits provided to people from habitat and regulating services.

Most official documents did not explicitly refer to the ES framework; four of 16 analysed documents did not refer to ‘ecosystem services’, ‘benefits’, or ‘human well-being’ terms, while eight documents did not use any of these terms more than twice. However, our study revealed that ten different ES were acknowledged in the literature about the CNP. These included food from agriculture, food from aquaculture, food from mushroom picking, habitats for species, soil erosion control, air purification, and water purification, nature recreation activities, aesthetic value and tranquillity of nature, as well as environmental education and scientific interests.

#### 4.1.2. Socio-cultural values of ecosystem services

The qualitative content analysis in the previous section revealed a general consideration of ES with regard to the importance of ecosystems functions, while a more comprehensive and differentiated understanding of people’s values with regard to ES was lacking. The survey results presented in this paragraph are meant to fill this gap by highlighting the appreciation of ES by different groups of the local society.

Firstly, local residents were showed to be broadly aware of the multifunctionality of the CNP in terms of the provision of diverse ES (Table 1). The most widely recognised benefits were air purification (93.4%) and habitat for species (91.8%), while the awareness for the provision of other services, such as water purification (59.2%), food from agriculture (68.8%), and food from aquaculture (75.2%) was considerably lower. What stands out is a high appreciation of the ES that were not fully recognised. For instance, although water purification was the least recognised service in the free listing of the survey (59.2%), it was still considered very important for people’s individual well-being (0.85 on a scale from 0 to 1). Similarly, food from agriculture was the second least recognised ES (68.8%), but still considered quite relevant (0.78). Regulating ES were considered most important for the local population (0.86), with air purification (0.88) and soil erosion control (0.86) rendering the highest importance levels overall. Cultural ES (0.83), follow the regulating group, were given stronger importance in environmental education and science (0.87). Provisioning ES resulted in the lowest value on average (0.66).

**Table 1.** Awareness (%) and appreciation of ecosystem services for personal well-being (mean value, normalized according to residents’ answers: (no = 1, minor = 2, significant = 3, strong importance = 4), and standardised using a minimum-maximum normalization [observation-min)/(max-min)] and considering 1 = 0 and 4 = 1)

Groups of Ecosystem Services	Ecosystem services	Awareness (%)			Importance for personal well-being (0–1)		
		Yes	No	don’t know	Mean for single ES	SD for single ES	Average of ES groups
Provisioning	Food from agriculture	68.8	11	20.2	0.78	0.29	0.66
	Food from aquaculture	75.2	12	12.7	0.66	0.29	
	Food from mushrooms picking	89.8	3.2	7.1	0.54	0.30	
Regulating	Habitat for species	91.8	3.2	5	0.85	0.23	0.86
	Soil erosion control	87	4.2	8.8	0.86	0.23	
	Air purification	93.4	1.5	5.1	0.88	0.20	
	Water purification	59.2	9.3	31.5	0.85	0.25	
Cultural	Nature recreation activities	85	5.8	9.2	0.82	0.23	0.83
	Aesthetic value and tranquillity of nature	81.2	5.7	13.2	0.81	0.25	
	Environmental education and science	89.7	1.6	8.7	0.87	0.21	

We disaggregated the survey data with regard to different social groups (Table 2) in order to reveal a more differentiated understanding of the ES values. For example, analysing the spatial component, respondents living in the park area tended to see habitat for species as less relevant than respondents not living within the National Park. Focusing on the age differences, the oldest respondents tended to provide lower scores for aesthetic value and tranquillity of nature, environmental education and science, as well as for all regulating services, all of which were much more highly valued by the youngest group of respondents. Differently, the youngest respondents tended to provide lower scores for nature recreation activities. A different pattern was only observed for food from mushroom picking, which was least appreciated by the mid-range age classes (45–54 and 55–64 years).

**Table 2.** Appreciation of ecosystem services for personal well-being for different groups of stakeholders (mean value, normalized according to residents’ answers: (no = 1, minor = 2, significant = 3, strong importance = 4), and standardised using a minimum-maximum normalization [observation-min)/(max-min)] and considering 1 = 0 and 4 = 1). Significant associations between stakeholders’ characteristics and ecosystem services perceived values by respondents and determined by Pearson chi-square test (values in bold are significance level<0.05)

			**Provisioning**			**Regulating**				**Cultural**		
			Food from agriculture	Food from aquaculture	Food from mushroomS picking	Habitat for species	Soil erosion control	Air purification	Water purification	Nature recreation activities	Aesthetic value and tranquillity of nature	Environmental education and science
**Personal Characteristics**	Gender	*Male (N = 190)*	0.74	0.64	0.54	0.85	0.84	0.86	0.82	0.80	0.81	0.84
		*Female (N = 175)*	0.82	0.68	0.55	0.85	0.88	0.90	0.88	0.84	0.82	0.90
		*X^2^ value*	**9.658**	3.000	2.289	6.810	3.478	4.940	**15.546**	**9.372**	7.066	6.071
		*X^2^ significance*	**0.022**	0.392	0.515	0.078	0.324	0.176	**0.001**	**0.025**	0.070	0.108
	Age	*18–24 (N = 28)*	0.70	0.61	0.70	0.94	0.97	0.97	0.88	0.74	0.88	0.94
		*25–34 (N = 55)*	0.82	0.69	0.61	0.86	0.84	0.89	0.89	0.84	0.82	0.87
		*35–44 (N = 67)*	0.84	0.72	0.55	0.90	0.89	0.88	0.85	0.84	0.83	0.86
		*45–54 (N = 70)*	0.80	0.62	0.48	0.83	0.85	0.89	0.86	0.85	0.85	0.90
		*55–64 (N = 54)*	0.69	0.62	0.47	0.84	0.80	0.84	0.80	0.77	0.79	0.80
		*65–74 (N = 48)*	0.79	0.70	0.53	0.80	0.87	0.94	0.89	0.86	0.81	0.89
		*>75 (N = 42)*	0.73	0.61	0.56	0.79	0.81	0.80	0.75	0.77	0.67	0.82
		*X^2^ value*	**35.349**	**35.156**	**50.519**	**34.858**	**37.384**	**53.591**	**53.325**	**44.272**	**42.778**	**37.808**
		*X^2^ significance*	**0.009**	**0.009**	**0.000**	**0.010**	**0.005**	**0.000**	**0.000**	**0.001**	**0.001**	**0.004**
	Residence	*Sabaudia (N = 243)*	0.76	0.64	0.54	0.84	0.85	0.87	0.82	0.83	0.83	0.87
		*San Felice Circeo (N = 122)*	0.80	0.70	0.54	0.88	0.87	0.90	0.90	0.80	0.79	0.87
		*X^2^ value*	3.875	4.444	1.354	7.308	4.773	6.919	**10.284**	5.757	1.864	0.525
		*X^2^ significance*	0.275	0.217	0.716	0.063	0.189	0.075	**0.016**	0.124	0.601	0.913
	Education	*Low (N = 76)*	0.79	0.68	0.57	0.83	0.85	0.87	0.87	0.78	0.79	0.84
		*Medium (N = 169)*	0.76	0.65	0.53	0.85	0.84	0.88	0.83	0.79	0.80	0.87
		*High (N = 120)*	0.80	0.65	0.54	0.86	0.88	0.90	0.86	0.88	0.84	0.89
		*X^2^ value*	3.163	9.768	3.542	11.139	8.521	2.352	3.499	**15.510**	**13.341**	3.337
		*X^2^ significance*	0.788	0.135	0.738	0.084	0.202	0.885	0.744	**0.017**	**0.038**	0.766
**Proximity to the Park**	House	*Park Area (N = 228)*	0.78	0.67	0.52	0.83	0.86	0.87	0.83	0.82	0.81	0.86
		*Outside (N = 137)*	0.77	0.64	0.58	0.89	0.85	0.90	0.87	0.81	0.82	0.88
		*X^2^ value*	1.028	4.625	4.827	**8.137**	7.750	3.462	2.607	6.526	0.156	1.232
		*X^2^ significance*	0.794	0.201	0.185	**0.043**	0.051	0.326	0.456	0.089	0.984	0.745
	Work	*Park Area (N = 111)*	0.80	0.65	0.49	0.84	0.85	0.86	0.82	0.82	0.81	0.85
		*Outside (N = 254)*	0.77	0.66	0.57	0.86	0.86	0.89	0.86	0.82	0.81	0.87
		*X^2^ value*	2.354	1.159	5.439	2.316	7.769	3.260	3.857	3.647	0.971	1.074
		*X^2^ significance*	0.502	0.763	0.142	0.510	0.051	0.353	0.277	0.302	0.808	0.783
	House and Work	*Park Area (N = 89)*	0.82	0.67	0.49	0.84	0.88	0.88	0.83	0.84	0.84	0.87
		*Outside (N = 276)*	0.77	0.65	0.56	0.85	0.85	0.88	0.85	0.81	0.80	0.87
		*X^2^ value*	4.086	1.223	4.710	3.192	**13.849**	0.298	4.391	2.540	5.299	1.091
		*X^2^ significance*	0.252	0.748	0.194	0.363	**0.003**	0.960	0.222	0.468	0.151	0.779
**Economic Activities**	Agriculture	*Yes (N = 36)*	0.81	0.65	0.53	0.84	0.81	0.87	0.88	0.81	0.81	0.88
		*No (N = 329)*	0.77	0.66	0.54	0.85	0.86	0.88	0.84	0.82	0.81	0.87
		*X^2^ value*	1.893	7.182	1.056	0.376	4.383	2.805	3.075	4.409	1.299	0.941
		*X^2^ significance*	0.595	0.066	0.788	0.945	0.223	0.423	0.380	0.221	0.729	0.816
	Fishing	*Yes (N = 5)*	0.96	0.96	0.96	0.93	0.96	0.96	0.96	0.96	0.90	0.96
		*No (N = 360)*	0.78	0.65	0.54	0.85	0.86	0.88	0.85	0.82	0.81	0.87
		*X^2^ value*	2.270	**8.787**	**17.818**	4.373	0.952	0.619	0.956	2.143	0.745	0.859
		*X^2^ significance*	0.518	**0.032**	**0.000**	0.224	0.813	0.892	0.812	0.543	0.863	0.835
	Industry and Crafts	*Yes (N = 32)*	0.76	0.65	0.48	0.78	0.81	0.83	0.82	0.85	0.83	0.85
		*No (N = 333)*	0.78	0.66	0.55	0.86	0.86	0.89	0.85	0.81	0.81	0.87
		*X^2^ value*	2.098	0.678	6.314	**11.281**	6.083	**10.816**	5.029	1.098	0.871	5.832
		*X^2^ significance*	0.522	0.878	0.097	**0.010**	0.108	**0.013**	0.170	0.778	0.832	0.120
	Building	*Yes (N = 4)*	0.64	0.59	0.56	0.83	0.83	0.83	0.83	0.77	0.63	0.83
		*No (N = 361)*	0.78	0.66	0.54	0.85	0.86	0.88	0.85	0.82	0.82	0.87
		*X^2^ value*	**8.766**	2.568	1.029	1.019	1.096	1.274	1.408	0.431	**9.780**	1.001
		*X^2^ significance*	**0.033**	0.463	0.794	0.797	0.778	0.742	0.704	0.934	**0.021**	0.801
	Wholesale and retail trade	*Yes (N = 23)*	0.90	0.85	0.59	0.87	0.84	0.91	0.92	0.85	0.85	0.90
		*No (N = 342)*	0.77	0.64	0.54	0.85	0.86	0.88	0.84	0.82	0.81	0.87
		*X^2^ value*	4.750	**12.195**	1.284	0.799	5.621	1.000	2.206	2.475	1.489	1.208
		*X^2^ significance*	0.191	**0.007**	0.733	0.850	0.132	0.801	0.531	0.480	0.645	0.751
	Hotel and catering sector	*Yes (N = 23)*	0.71	0.59	0.49	0.79	0.86	0.84	0.80	0.75	0.85	0.84
		*No (N = 342)*	0.78	0.66	0.55	0.85	0.86	0.88	0.85	0.82	0.81	0.87
		*X^2^ value*	**21.925**	**12.460**	**10.386**	6.154	2.009	5.585	6.095	**13.368**	0.628	6.674
		*X^2^ significance*	**0.000**	**0.006**	**0.016**	0.104	0.571	0.134	0.107	**0.004**	0.890	0.083
	Real estate activities	*Yes (N = 12)*	0.85	0.60	0.57	0.89	0.82	0.80	0.74	0.79	0.84	0.88
		*No (N = 353)*	0.78	0.66	0.54	0.85	0.86	0.88	0.85	0.82	0.81	0.87
		*X^2^ value*	1.698	1.274	2.173	3.137	**8.706**	5.601	7.536	3.381	1.850	4.524
		*X^2^ significance*	0.637	0.735	0.537	0.371	**0.033**	0.133	0.057	0.336	0.604	0.210
	Professional, scientific, and technical activities	*Yes (N = 36)*	0.72	0.69	0.50	0.86	0.86	0.86	0.85	0.79	0.76	0.84
		*No (N = 329)*	0.78	0.65	0.55	0.85	0.86	0.88	0.85	0.82	0.82	0.87
		*X^2^ value*	1.890	1.595	1.175	6.704	2.876	4.564	0.548	2.995	2.226	3.688
		*X^2^ significance*	0.595	0.660	0.759	0.082	0.411	0.207	0.908	0.392	0.527	0.297
	Public administration and defence	*Yes (N = 59)*	0.70	0.66	0.51	0.84	0.83	0.83	0.76	0.80	0.77	0.83
		*No (N = 306)*	0.79	0.65	0.55	0.85	0.86	0.89	0.87	0.82	0.82	0.87
		*X^2^ value*	**20.510**	**11.996**	**8.330**	5.747	2.739	**11.183**	**10.668**	5.238	**10.420**	2.874
		*X^2^ significance*	**0.000**	**0.007**	**0.040**	0.125	0.434	**0.011**	**0.014**	0.155	**0.015**	0.416
	Education	*Yes (N = 44)*	0.75	0.63	0.59	0.79	0.85	0.90	0.85	0.81	0.81	0.88
		*No (N = 321)*	0.78	0.66	0.54	0.86	0.86	0.88	0.85	0.82	0.81	0.87
		*X^2^ value*	**8.185**	0.968	2.796	5.054	6.740	1.814	4.380	7.315	3.181	2.964
		*X^2^ significance*	**0.042**	0.829	0.424	0.168	0.081	0.612	0.223	0.062	0.365	0.397
	All Other Activities (Communication, Health, Sport, etc.)	*Yes (N = 86)*	0.74	0.60	0.48	0.87	0.89	0.89	0.85	0.83	0.83	0.90
		*No (N = 279)*	0.46	0.34	0.23	0.51	0.51	0.55	0.52	0.48	0.48	0.52
		*X^2^ value*	3.488	7.452	4.785	1.969	6.448	4.977	0.647	2.800	**8.787**	**9.207**
		*X^2^ significance*	0.322	0.059	0.188	0.579	0.092	0.174	0.886	0.423	**0.032**	**0.027**

In terms of gender difference, female respondents tended to give higher scores for all the ES compared to male respondents, with statistical significance for food from agriculture, water purification, and nature recreation activities. Table 2 shows that there is also a different appreciation based on the respondents’ educational level. In particular, as the level of education increased, so did the value assigned to nature recreation activities and aesthetic values and tranquillity of nature augmented.

When looking at business sectors, we found statistically significant differences in respondents engaged in different working areas. Respondents from the industry and crafts sectors tended to give lower importance to habitat for species and air purification, while workers in the building sector tended to give lower scores for food from agriculture, aesthetic values and tranquillity of nature. Differently, respondents working in fishing and aquaculture more strongly appreciated food from aquaculture and food from mushroom picking, while workers in the hotel and catering sectors tended to give lower scores for food from agriculture, food from aquaculture, and food from mushroom picking. Moreover, although these workers gave slightly lower values for nature recreation activities, 74% of them perceived these benefits as ‘quite important’. What is striking in Table 2 is the general pattern of lower appreciation for the public administration and defence area, with statistically significant data for all the provisioning services, aesthetic values and tranquillity of nature, and air and water purification. Finally, we found differences in the valuation of respondents from other activities, such as health, assistance, and sporting sectors. These respondents perceived aesthetic values and tranquillity of nature and environmental education and science as very important benefits.

### 4.2. Procedural justice

#### 4.2.1. Participation

The qualitative content analysis explored decision-making processes in order to understand the involvement of different stakeholders in the CNP management. The most interesting result emerged from the strong emphasis in the CNP-Plan regarding procedural justice as the plan is meant to guarantee – in its development, definition, and its subsequent implementation – a shared and participative approach with local administrations and stakeholders, at any level interested in the CNP territory and its surroundings (Ente Parco Nazionale del Circeo 2011a). The CNP-Plan was developed through an interactive process, conducted by groups of experts from different disciplines and with the involvement of the inhabitants, their associations, institutional bodies, businesses, and civil society located within the CNP. The process constituted a continuous and open process of governance, aimed at building the general framework of the CNP area, defining conservation goals, but also addressing the needs of local people. In detail, in 2017, the CNP Authority organised six open thematic forums intended for different local groups (associations, traders and hoteliers, bathing establishments, sports associations, tour operators, farmers and breeders) for a total of about 120 participants; four participatory working groups with about 30 participants of the municipalities representatives and farmers and breeders associations; and three open assembly meetings for the presentation of the preliminary and the completed CNP-Plan, with a turnout of about 90 people (Parco Nazionale del Circeo 2020). Yet, the identification of interlocutors from various sectors involved in the planning process was not explained in the CNP-Plan, leaving the reference to stakeholders’ needs vague. Moreover, when contrasted with the survey results on local people’s perception of the participatory process, the level of procedural justice seems to be lower compared to what we revealed from official policy documents. This seems primarily to be related to a lack of information and communication.

#### 4.2.2. Information and communication

Three items on the questionnaire examined respondents’ level of participation, their perception about CNP information and communication strategies, and their level of confidence in CNP Authority. Despite the rich participatory process described above and the attempt to incorporate different stakeholders and their interests, our questionnaire results indicated that 75% of the local respondents were not aware of these participatory initiatives, while 12% of the respondents had engaged in the development of the CNP-Plan at least at one of the stages of the participatory process. Among the non-participants, 64% declared they would have been interested in participating if they had been properly informed, while only 11% of the respondents stated they would not have been interested in participating in the CNP-Plan development at all. When further asked to evaluate the communication strategy for the CNP initiatives, respondents highlighted the low level of information and lacking diversity of communication. Our findings also revealed a relatively low level of confidence in the general management capacities by the CNP Authority, including decision-making processes, but also the resolution of problems and tensions with citizens. While 34% indicated a high (27%) or very high (7%) level of confidence in the park management, 66% of the respondents indicated low (39%) or very low levels of confidence (27%). Again, the social group with the lowest levels of confidence in the park management was the respondents living in the park area (70%), confirming the findings under the recognition dimension which underlined a spatial injustice.

### 4.3. Distributional justice

#### 4.3.1. Social inequalities

As underlined by results about the recognitional justice, strategies adopted by the CNP neither considered whether they generated burdens or disadvantages in relation to different ES for the community as a whole, nor whether this applied for some societal groups. Addressing our goal to examine potential negative impacts on citizen groups due to the protection regime of the park, we asked whether the CNP impacted survey respondents’ well-being positive or negatively. To this question, more than half (56%) of the respondents defined the general impact as positive, 37% as neutral and only 7% as negative. Yet, in accordance with the documents review, most of the respondents who stated to be negatively affected live and/or work within the CNP boundaries (live 72%, work 44.5%, live and work 37%) and perceive conservation to limit the development of their activities (37.6% in Table 3).

**Table 3.** The questionnaire asked whether and how the Circeo National Park have impacted survey respondents’ well-being. Here we resume the main reasons for respondents (7%) who defined the presence of the park as negative for their life and activity

Reasons for the negative impact	%
Limitations for production activities and economic development	37.6
Inefficiency of Park Authority	20.8
High bureaucracy for permissions (ex: tree cutting)	19.4
Sense of prohibition and not protection	18.5
No maintenance (fences, roots, trees, drains, paths, walkways, etc.)	15.0
Limitations to urbanization	14.4
Privatization of Paola Lake	5.9
Inability of Park Authority to enforce regulations (ex: illegal cut and waste in the park area)	3.9
No animals control	3.6
No answer	19.0

The first group that sees itself negatively affected by the CNP are people working in the tourism sector. The intensification of tourism, which includes installations, trampling, and parking, has been documented as the principal cause of severe degradation of the coastal dune system and other ecosystems, which reduces their capacity to provide ES, such as soil erosion control (Acosta et al. 2000; Aretano et al. 2017). In response, the Park Authority adopted different conservation strategies, such as the installation of walkways to reach the beach, as well as regulatory limitations for new buildings and the expansion of existing buildings, which have contributed to the stabilization of coastal ecosystems (Aretano et al. 2017), and augmented cultural ES. Yet, the tourism sector perception was that they bore the burdens of these measures (for instance, losing earnings for a smaller number of tourists presence or due to limitations on accommodation capacity).

The other social group that felt most negatively affected were local farmers situated within the park area. The primary goal of the CNP to preserve biodiversity, for instance, by providing suitable living and nursing places for wild species, was assumed to stand in opposition to the farmers’ interests. This group pinpointed the negative effects of the CNP in relation to the ‘limitations for production activities and economic development’ (Table 3). The CNP regulation disciplines agricultural and pastoral activities in the park area, for instance, requiring farmers to adopt organic farming methods, while intensive agriculture was developed in the direct surroundings of the park, creating an important discrepancy and inequality between farmers inside and outside the CNP. In the general oriented reserves (see Appendix VII for more details), only pesticides using biological and integrated control techniques are permitted, except with specific authorization issued by the Park Authority for serious and demonstrable reasons. In these areas of protection, the use of pesticides and chemical fertilizers is permitted. Furthermore, the use of permanent greenhouses is allowed only if the farm surface and structures concerned comply with certain parameters relating to dimensions, boundaries, roofing material, or irrigation systems, while the creation of new structures is only allowed for organic production. This is in line with the negative impacts stated by most respondents, primarily related to injustices in the distribution of benefits from provisioning ES, namely food production. This perception goes hand-in-hand with a general sense of prohibition and regulatory limitations for economic development, and especially for agricultural production. It has further been related to inefficiencies in the CNP Authority to make decisions and high bureaucratic burdens for permissions (Table 3). Another problem frequently stated by local farmers regards the expansion of wild boars causing damage for local crop production, and together with fallow deer, they were also reported as a risk for road traffic. In response to these problems, the Park Authority proposed the creation of the ‘Park Mark’ as an identity, characterization, and promotion tool for the products and services of the territories within the CNP (Ente Parco Nazionale del Circeo 2011a, 2011b). The goals of the ‘Park Mark’ are to promote and certify the environmental quality of organic farming and breeding, fishing and aquaculture, hotel and non-hotel structures, as well as touristic services. While this could be a possible way to give more visibility to the economic activities within the CNP territories, compensating for some distributive inequalities, it will probably not solve them.

#### 4.3.2. Spatial-temporal burden shifting

Our last objective of the distributive section was to identify how conservation policies generated social inequalities between different stakeholders’ groups, in particular according to the different land use within and outside the park area. With the aim of making the socio-economic activities compatible with conservation objectives, the Park Authority, through the CNP-Plan and the zoning map (Ente Parco Nazionale del Circeo 2011c), regulates the general organization of the territory and defines restrictions, rules, destinations for public and private use. For instance, agricultural, pastoral, as well as fishing activities, are allowed according to the traditional uses or according to methods of organic farming, in compliance with the criteria established by the Park Authority. Finally, in the areas of economic and social promotion, activities in line with establishing the park and aimed at improving the socio-cultural life of local communities are allowed, with the highest enjoyment of the park by visitors. Nevertheless, the CNP surrounding areas (Figure 2) have undergone an important intensification process that stands in sharp contrast to the activities permitted within the park, and which can also be related to burden shifting, i.e. negatively affecting activities within the PA. Intensive agriculture and the use of pesticides have led to the pollution of the soil and consequently the ground and surface waters (Sappa et al. 2005; Manca 2014). Additionally, groundwater pumping to supply water for intensive agriculture has caused the depletion of groundwater sources; especially in springtime, agricultural production is intense and the strain on groundwater resources is large (Manca 2014). The absence of a regional water regulation (Manca 2014) and uncontrolled withdrawals have been reported as endangering the natural system, amplifying the depletion of groundwater and degradation of the quality of underground water resources due to the progressive increase in the phenomenon of seawater intrusion (Sappa et al. 2005; Manca 2014). Another damaging impact was the pollution of the CNP lakes, resulting in problems for aquaculture production in Paola Lake. Together with an increase in temperature, pollution and nutrient loads from intensive agriculture and civil waste decreased the oxygen of the waters, creating a habitat crisis. This caused the disastrous death of fish in July 1979, and other similar phenomena, though less intense, in 2003 and 2015 (Parco Nazionale del Circeo 2018c). These phenomena negatively affect food production from aquaculture, reducing the economic benefits for the owners of Paola Lake. Yet, as shown in Tables 3, 5.9% of the respondents who declared a negative impact, perceived the privatization of Paola Lake as an injustice.

Table 3: *The questionnaire asked whether and how the Circeo National Park have impacted survey respondents’ well-being. Here we resume the main reasons for respondents (7%) who defined the presence of the park as negative for their life and activity.*

## 5. Discussion

One of the principal recommendations of the Millennium Ecosystem Assessment for PAs was to develop policies and other effective means based on the benefits and values of the services PAs provide (MA, 2005). Embracing an ES approach can help conservation strategies to integrate multiple policy objectives, including diverse social interests in parallel with preserving ecosystem integrity and health (Agrawal and Redford 2009; Dawson et al. 2017; García-Llorente et al. 2018). However, although PAs shall be designed and managed to provide benefits to society, they are often not understood in that sense (Palomo et al. 2011), ES are still not explicitly considered in many PAs (Geijzendorffer et al. 2017), and conservation approaches are still primarily driven by an exclusionary model, in which people are separated from nature (Martin et al. 2016). The CNP mirrors these problems, with very few conservation objectives that consider benefits provided to people or quantify values from the ecosystems. We analysed the CNP-Plan and the Environmental Strategic Assessment documents. The former regulates the use of the territory, defining restrictions and rules and establishing criteria for interventions on flora and fauna. The latter aims at integrating environmental considerations into development plans to improve overall decision-making quality. Despite their participatory and inclusive nature, we found an important lack of explicit inclusion of the human dimension (*e.g*. beneficiaries, peoples’ values and needs) and a limited understanding of the socio-economic aspects of PA. Nevertheless, implementing an ES framework may extend conservation objectives beyond moral and intrinsic values, i.e. to protect biodiversity for its own sake (Cowling et al. 2008; García-Llorente et al. 2018), considering in addition both relational values that emotionally attach people to the environment, and assigned values, including purely instrumental benefits nature provides to people, for instance, revenues from nature tourism (Arias-Arévalo et al. 2017; Chan et al. 2018). Moreover, conservation strategies that consider social values can result in examining perceived environmental qualities in local contexts, identifying priority ES for management objectives, recognising vulnerable stakeholders, decreasing conflicts over land use, and increasing community engagement in cost-sharing, volunteerism, and environmental management (Tyrväinen et al. 2007; Bryan et al. 2010, 2011; Iniesta-Arandia et al. 2014). ES, thus, could provide an anthropocentric approach for pursuing preservation goals and, simultaneously, improving the social acceptance for conservation, allowing a better understanding of PAs in their social-ecological complexity and under a more equitable point of view.

### 5.1. Recognising diverse values for just PAs planning

As observed elsewhere, the lack of awareness for ES does not necessarily mean that specific benefits are of lower or of no value for local communities (*cf*. Asah and Blahna 2020). Our results about the socio-cultural assessment underlined that social actors’ different values of ES need to be recognised (Cáceres et al. 2015), in order to uncover potential conflict lines and perceived injustices in PAs strategies. However, our study revealed a general mismatch between the CNP goals and people’s values, which may indicate perceived injustices and a latent conflict (Hanaček et al. 2021), and which might undermine an efficient protection regime. For instance, in terms of provisioning ES, stronger importance was given in the CNP policies than by the respondents. Similarly, regulating ES, which play a minor role in the policies for the interests of the society, were considered most important for the local population. Next to the generic biodiversity conservation objectives, PAs should improve the understanding of the ES provided to human well-being (García-Llorente et al. 2018), especially the needs of those living and within the park boundaries that do not seem to be properly acknowledged. Our results indicate that there may be an ongoing shift regarding the relevance of certain ES, as age was a significant factor influencing the appreciation of ES, with the main differences being between young and old respondents. In fact, while the CNP policies well reflect the interests of older people, they didn’t consider likewise the preference of younger people. If younger people maintain their preference as they grow older, and if CNP policies are not able to adapt accordingly, this will likely lead to a stronger fissure in recognition of justice. The disaggregated analysis further indicates a potential temporal shift in ES values widening the gap in recognition justice. Similar to observations by Oteros-Rozas et al. (2014), while elders enjoy recreation, the younger more strongly value regulating services (potentially related to more formal environmental education), which is, however, insufficiently reflected in CNP policies. In effect, the human use of ecosystems and their services also raises fundamental questions of intra-generational justice; and an empirical investigation of ES justice must also consider ES trade-offs between the current and future generations (Glotzbach 2013). A key policy priority should therefore be to plan for long-term conservation goals, involving, young people in the future CNP-Plan together with traditional economic stakeholders. This is a critical finding as recognising stakeholders’ needs can be assumed as a prerequisite for fair planning processes. Some examples related to the working sector also highlighted the importance of framing perceived injustices within a temporal perspective. For instance, the tourism sector can be primarily assumed to have followed an unsustainable business model based on degradation and this required intervention in the form of regulations. At the same time, farmers can be assumed to be affected because their problems were not properly addressed by the park management. Additionally, our disaggregated results about gender mirror previous findings on gender-specific ES preferences, though recognition injustices were not evident. For instance, in different studies, such as Martín-López et al. (2012), Oteros-Rozas et al. (2014), or Asah and Blahna (2020), females typically perceived regulating and cultural services as more valuable and exhibited more environmental behaviour than men, while males mostly perceived provisioning services. Langemeyer et al. (2018a) assumed that female socialization might positively influence the awareness of benefits from nature and confirmed the view that different values might be rooted in gender differences (Dietz et al. 2002). Nevertheless, so far, gender differences in ES values have still not properly been explained and require additional research, also adopting a justice lens. Lastly, the disaggregated recognition of different societal groups’ values allowed us to underline the interdependence between ES and some economic activities, such as the case of fishermen and tourism operators. However, this was not true for the agriculture sector: responses by farmers were more heterogeneous and not statistically significant. Although these stakeholders were partially accounted for in the CNP documents, their needs and preferences do not seem to be properly acknowledged. This finding is consistent with that of Martín-López et al. (2019) in which some local actors (*e.g*. farmers and fishermen) were strongly dependent on ES, but their representation in decision-making was low, which affected both procedural and distributional justice.

### 5.2. Socio-spatial implications of unequal distributions of ES benefits and burdens

The intensive land use around many PAs emphasises another important issue: PAs cannot be managed as isolated and static entities (Palomo et al. 2013). In the CNP, this was primarily the case for farmers from within the PA and identified by the results of all three spheres of justice as the group that felt unequally and negatively treated compared to their peers outside the PA. They also suffered from burden shifting and environmental impacts caused outside the PA boundaries. At the same time, CNP farmers can be assumed critical for any conservation strategy as they act as important stewards of the cultural landscapes within the CNP. If they remain in a state of latent conflict, opposition to the conservation efforts may raise and its effectiveness decline (Palomo et al. 2011; Hanaček and Rodríguez-Labajos 2018). ES have effects across different spatial scales which must be accounted for, both, regarding the distribution of benefits, as well as potential costs for their production and beneficiaries (Ernstson 2013; Jax et al. 2013). As a matter of fact, the distributive analysis highlighted different inequalities due to negative impacts of the CNP policies, trade-offs between nature conservation and benefits to people, and different planning interventions and strategies in adjacent territories. These observations call for a dedicated spatial approach to ES justice in PAs management that goes beyond PAs boundaries. The negative effects of land use change and intensification occurring outside a PA, which create border effects that impinge upon the ES delivery within a PA have been described elsewhere (García-Llorente et al. 2018). It has also been commonly found that perceived injustices are primarily attributed to bad PA management rather than to ecologically inappropriate activities by farmers in areas surrounding a PA (*cf*. Turkelboom et al. 2018; Langemeyer et al. 2018b). That means a spatial ES justice approach needs to align activities in the surrounding of a PA with the protection goals and regulations within a PA in order to resolve injustices. Regulations should consider the land uses surrounding a PA, and the extent of its isolation from or connectivity to other natural areas (Hockings et al. 2006). Management should confront the problem of managing the entire complexity of social-ecological landscapes, which often consists of interactions among different habitats and ecosystems, and integrating phenomena across multiple spatial, temporal and organizational scales (Petrosillo et al. 2009). Still, PAs are too often not understood and managed to provide benefits to society (Palomo et al. 2011), and tend to focus on their main mission to preserve biodiversity. Incorporating the idea of ES and EJ into conservation may help to identify and overcome conflicts given in PAs and increase the acceptance of conservation measures.

### 5.3. Procedural justice is more than participation

Over the last few decades, authors have argued that sustainable management of natural resources cannot be achieved without the involvement of the affected communities, participating in the management of PAs (*e.g*. Palomo et al. 2011; Gustavsson et al. 2014; Setti et al. 2016). Stakeholder engagement has been seen as a way to guarantee that plural values held by stakeholders are considered (Shrader-Frechette 2002; Kenter 2016), thereby linking recognition with procedural justice consideration. Moreover, embracing an ES approach in PAs management requires engaging people. Indeed, different authors (*e.g*. Chan et al. 2012; Bennett et al. 2015; Sattler et al. 2018), have underlined the need to include participatory approaches in ES governance in order to identify priority ES on the basis of stakeholders’ inputs and viewpoints. However, the CNP, as analysed here, follows a typical top-down decision-making and management process, which tends to overlook local practices and neglect local interests, thus enhancing the conservation versus development model dichotomy and bringing about conflicts that undermine conservation goals (West et al. 2006; Palomo et al. 2011), which tends to overlook local practices and neglect local interests, thus enhancing the conservation versus development model dichotomy and bringing about conflicts that undermine conservation goals. This underlines the importance of streamlining participatory processes and improving communication strategies. As highlighted by Buono et al. (2012), ineffective provision of information and failed communication can hamper the acceptance of decisions made by PAs managers, similar to the effect of inappropriate participation procedures. Indeed, this has been confirmed also by our findings, showing a low level of information and communication perceived by respondents and, consequently, a low level of trust in the management of the CNP Authority. Effective management approaches should consider involving local communities and should at least record the quality of relationships between PAs managers and local people (Hockings et al. 2006). PAs policies should include the development of a common understanding of ES and agreements that are reached, which consider the interests of all stakeholder groups, especially when many actors with their different perceptions and needs are involved (Hauck et al. 2013; Fabinyi et al. 2014), with particular emphasis on the most vulnerable groups – which in our study and across all three dimensions of justice, were people living within the park boundaries. Succinctly, procedural justice requires approaches beyond participation and a critical understanding of the framing of decision-making (Langemeyer and Connolly 2020). Where our study has primarily highlighted the importance of information and communication (in line with Connolly and Steil 2009), others have indicated the need to create equitable spaces of engagement (Martin et al. 2016; Nunan et al. 2018) and to strengthen measures that account for power relations in decision-making processes (Pascual et al. 2017; Dawson et al. 2018). Fair procedures are assumed to better integrate people’s needs and preferences, in terms of recognition, which ultimately is meant to lead to the just distribution of benefits and burdens generated by PAs.

## 6. Conclusions

The purpose of this study was to empirically investigate the case of a PA, combining the ES and EJ frameworks. The integrated ES and EJ approach applied in this study allowed us to gain a differentiated understanding of the distribution of benefits and burdens among different social groups and the underlying reasons for perceived injustices, which primarily lay in the lack of recognition of ES preferences and insufficient procedural justice, especially in terms of communication. If, on the one hand, the distributive issues are rooted in recognitional and procedural omissions, on the other, the lack of recognition of ES beneficiaries is strongly related to the dated vision of safeguarding biodiversity and excluding people from PAs in terms of procedural injustice. These findings have significant implications for the understanding of how future analysis about EJ cannot consider three classical dimensions separately. Moreover, our research further indicates that temporal and spatial dimensions of ES justice are interwoven within classical EJ dimensions; for instance, in the shifting preferences for ES over time indicated by our study, which have not yet been properly addressed by CNP management. The issue of spatial and temporal spheres is an intriguing one that should be usefully explored in further research. Finally, our study contributes in several ways to the understanding of possible perceived injustices and conflict lines in PAs and provides a basis for management decisions in developing fairer and more sustainable socioecological systems. For example, our findings suggest several courses of action for the CNP, such as the improvement of strategies for citizen’s involvement and effective communication, or more inclusive planning that takes into account people’s needs, as well as the areas surrounding the park. Along with conservation objectives, ensuring support for local communities should be a priority for all PAs.

## Supplementary Material

Appendices

## Note

1. We followed the ethical principles of anonymity, right to privacy, and informed consent of respondents (Bryman 2012). At the beginning of the questionnaire, we provided a short informative paragraph, in order to give sufficient information about the aim of the research, what respondents were being asked and how their responses would have been used. In the sheet, we guaranteed anonymity and compliance with the law on privacy of the survey, in as much as respondents were completely free to withhold their names and data collected have been treated in an aggregate manner. For more information, see the entire questionnaire in Appendix VI.
