# Supplementary material for: Ecosystem services and justice of protected areas: the case of Circeo National Park, Italy
Source: Ecosyst People (Abingdon). 2021 Jul 21;17(1):411–31. doi: 10.1080/26395916.2021.1946155 (PMC8315210; doi:10.1080/26395916.2021.1946155)

APPENDIX I – BODY AND TOOLS OF THE DECISION-MAKING AND MANAGEMENT PROCESSES, AND THEIR IMPLEMENTATION IN CNP

Body/tools	Description	Implementation in CNP
**Park Authority**	Institution with personalities of public law, legal and administrative headquarters in the territory of the Park and supervised by the Minister of the Environment. Composed by the President, the Governing Council, the Director, the Executive Council, the Auditors Council and the Park Community. Unlike the other figures, the Park Community is an advisory and proposing body, constituted by presidents of the Regions, presidents of the Provinces and mayors of the Municipalities of Park territories (these administrative figures are elected by local communities).	Since its establishment in 1933, Circeo National Park was managed by the State Forestry Corps. Despite the entry into force of the Italian Law 394/1991, only in 2005 a specific regulatory provision established the Park Authority. Moreover, the President and the Governing Council were elected only in 2007 and the Director in 2008. The Executive Committee has not yet been established, just as the Vice-President has not yet been appointed.
**Statute**	The Statute of the Park Authority, drawn up by the Governing Council and adopted by the Minister for the Environment, defines the internal organization, the modalities of popular participation, the forms of publicity of the documents.	CNP Statute was adopted with the ministerial decree in 2009.
**Regulation**	The Regulation of the Park, adopted by the Park Authority, regulates the exercise of the activities allowed within the territory of the Park, including agricultural and commercial activities, transport circulation, recreational and research activities, accessibility in the Park territory, prohibited activities, etc. The Park Regulation is approved by the Minister for the Environment, after consultation with the Council and after consulting the local authorities concerned. After its publication in the Official Gazette of the Italian Republic, the Municipalities are required to adapt their regulations to the Park Regulation.	CNP regulation was already adopted by the board of the Park authority in 2012 but will be approved by the Minister of the Environment only after the approval of the Park Plan.
**Park Plan (PP)**	The PP regulates general organization of the territory in areas characterized by different forms of use, enjoyment and protection; restrictions, rules, destinations for public and private use; systems of vehicular and pedestrian accessibility; systems of equipment and services for the management and social function of the Park, museums, visitor centres, information offices, camping areas, agritourism activities; and criteria for interventions on flora and fauna. The PP is draft by the Park Authority, adopted by the Region of competence, after having heard the local authorities and taken into consideration the observations to the Plan that can be presented by anyone (including individuals) in writing form. The plan is published in the Official Gazette of the Italian Republic and in the Official Bulletin of the Region and is immediately binding on administrations and private individuals.	PP of CNP has remained for years in preparation and discussion. In 2011 it was approved by the Governing Council but waited for the opinion of the Park Community. Finally, on 25th of July 2017, the Regional Council of Lazio adopted the PP and the Strategic Environmental Assessment (SEA). On November 2017, CNP presented to the Region 366 observations for the PP and 150 for the SEA, but, currently, the process for the final acceptance is stopped in the Region.
**Long-term Economic and Social Plan (E&S Plan)**	Proposed by the Park Community, the E&S Plan suggests the initiatives aimed at favouring the economic and social development of the collectivises residing within the Park and adjacent territories. The E&S Plan is submitted to the binding opinion of the Governing Council and is approved by the Region.	The draft of the E&S Plan was developed by the Park Community in the period 2002-2008, validated from a technical-administrative point of view, but not yet adopted.

APPENDIX II – ADDITIONAL INFORMATION ABOUT THE POPULATION AND ECONOMIC SECTORS IN THE CNP AREA

Population of Circeo National Park's Municipalities (Data source: ISTAT, 2018; Personal communication from the Municipalities of Latina, Sabaudia, and San Felice Circeo).

Municipality	Municipal population	CNP population	CNP population compared to municipal population (%)	CNP population compared to the total CNP population (%)
**Latina**	126.151	985	0.78	6.38
**Ponza**	3.348	0	0.00	0.00
**Sabaudia**	20.613	12.418	60.24	80.45
**San Felice Circeo**	10.025	2.032	20.27	13.16
**Total**	160.137	15.435	9.64	100.00

Number of local units of active enterprises for different economic activities (based on the Ateco 2007 classification of economic activities, which constitutes the Italian version of the European nomenclature Nace Rev.2) in the Municipalities of Sabaudia and San Felice Circeo (Data source: ISTAT, 2010; ISTAT, 2017a; ISTAT 2017b**) and Number of employed from Sabaudia and San Felice Circeo by sections of economic activity (Data source: ISTAT, 2011).*

Classification of economic activities (Ateco 2007)		Local Units		Employees	
		Sabaudia	San Felice Circeo	Sabaudia	San Felice Circeo
**A1**	Agriculture	679*	253*	1533	619
**A2**<br>**A3**	Forestry<br>Fishing	Data not available	Data not available		
**B**	Extraction of minerals from quarries and mines	1	0	1210	586
**C**	Industry and Crafts (transformation of raw materials)	105	57		
**D**	Supply of electricity, gas, steam, and air conditioning	5	1		
**E**	Water supply sewerage, waste management and remediation activities	3	2		
**F**	Buildings	113	79		
**G**	Wholesale and retail trade	369	226	1413	637
**I**	Hotel and catering sector	128	111		
**H**	Transport and storage	26	19	344	174
**J**	Information and communication services	29	7		
**K**	Financial and insurance activities	32	16	684	303
**L**	Real estate activities	51	24		
**M**	Professional, scientific, and technical activities	118	73		
**N**	Rental, travel agencies, business and support services	44	30		
**O**	Public administration and defence	2**	1**	2125	668
**P**	Education	12	4		
**Q**	Health and social assistance	76	26		
**R**	Artistic, sporting, and entertainment activities	21	26		
**S**	Other activities	64	51		

APPENDIX III – TYPOLOGY AND DESCRIPTION OF REFERENCES USED IN THE CASE STUDY ANALYSIS

Typology	Description	References
**Official Italian Laws**	Constitution of the Park	Gazzetta Ufficiale della Repubblica Italiana (2005). Decreto del Presidente della Repubblica 2005, n. 155. Istituzione dell'Ente Parco Nazionale del Circeo. Pdf file retrieved from: https://www.minambiente.it/sites/ default/files/dpr_04_04_2005_pn_circeo.pdf
	National framework law on protected areas	Gazzetta Ufficiale della Repubblica Italiana (2018). Legge 6 dicembre 1991, n. 394. Legge Quadro sulle Aree Protette. Pdf file retrieved from: http://www.gazzettaufficiale.it/eli/id/1991/12/13/091G0441/sg
	Approval of the Statute of the Circeo National Park	Ministero dell'Ambiente e della Tutela del Territorio e del Mare (2008). CD PNC 22 dicembre 2008, n. 27. Approvazione dello Statuto Ente Parco Nazionale del Circeo. Pdf file retrieved from: https://www.minambiente.it/sites/default/files/archivio/normativa/ statuto_ente_parco_circeo_22072009.pdf
	Report on the state of implementation of the law on protected areas	Ministero dell'Ambiente e della Tutela del Territorio e del Mare (2012). Relazione sullo stato di attuazione della legge n. 394 del 1991. Pdf file retrieved from: https://www.minambiente.it/sites/default/files/ archivio/allegati/trasparenza_valutazione_merito/ STATO%20DI%20ATTUAZIONE%20DELLA%20LEGGE% 206%20DICEMBRE%201991%20-%20anni%202011-2012.pdf
**Policy documents of the CNP**	Scheme of the CNP-plan	Ente Parco Nazionale del Circeo (2010). *Schema di Piano del Parco*. Pdf file retrieved from: http://www.parcocirceo.it/pagina.php?id=110
	Regulation of the Park	Ente Parco Nazionale del Circeo (2011a). *Piano del Parco Nazionale del Circeo. Regolamento*. Pdf file retrieved from: http://www.parcocirceo.it/pagina.php?id=110
	CNP-plan (volume 1-3 and implementing technical standards)	Ente Parco Nazionale del Circeo (2011b). *Piano del Parco Nazionale del Circeo. Relazione Generale –Tomo 1*.<br>Ente Parco Nazionale del Circeo (2011c). *Piano del Parco Nazionale del Circeo. Relazione Generale – Tomo 2*.<br>Ente Parco Nazionale del Circeo (2011d). *Piano del Parco Nazionale del Circeo. Relazione Generale – Tomo 3*.<br>Ente Parco Nazionale del Circeo (2012). *Piano del Parco Nazionale del Circeo. Norme tecniche di attuazione*.<br>All Pdf files retrieved from: http://www.parcocirceo.it/pagina.php?id=110
	Map of the zoning	Ente Parco Nazionale del Circeo (2011e). Tavola 2 – Carta della zonizzazione. Pdf file retrieved from: http://www.parcocirceo.it/pagina.php?id=110
	Map of ecosystem services values	Fondo Europeo Agricolo per lo Sviluppo Rurale (2013). *Programma di Sviluppo Rurale 2007-2013 – Carta della valenza dei servizi ecosistemici*. Pdf files retrieved from: http://www.parcocirceo.it/pagina.php?id=112 (Tav.I.1 and Tav.I.2)
	Strategic environmental assessment	Parco Nazionale Del Circeo (2016). *Piano Del Parco – Valutazione Ambientale Strategica – Rapporto Ambientale 12/2016*. Pdf file retrieved from: http://www.parcocirceo.it/pagina.php?id=110
**Websites**	Official website of the Park	Parco Nazionale del Circeo (2018a). *Parco Nazionale del Circeo*. Online: http://www.parcocirceo.it/
	Online grey literature of the Park in the period January 2016-December 2018 (news and press review)	Parco Nazionale del Circeo (2018b). *News*. Online: http://www.parcocirceo.it/nov.php<br>Parco Nazionale del Circeo (2018c). *Rassegna stampa*. Online: http://www.parcocirceo.it/rassegna.php

Typology	Description	References
**Secondary data**	Data about Municipal populations	ISTAT – Istituto Nazionale di Statistica (2018). Popolazione residente al 1° gennaio 2018. Dati scaricati da: http://dati.istat.it/Index.aspx?DataSetCode=DCIS_POPRES1
	Data of the Park population	Personal communication from the Municipalities of Latina, Sabaudia, and San Felice Circeo
	Data of active enterprises and employed for different economic activities	ISTAT – Istituto Nazionale di Statistica (2010). Censimento Agricoltura 2010. Dati scaricati da: http://dati-censimentoagricoltura.istat.it/Index.aspx?lang=it<br>ISTAT – Istituto Nazionale di Statistica (2011). Censimento Popolazione Abitazioni 2011. Dati scaricati da: http://dati-censimentopopolazione.istat.it/Index.aspx?lang=it<br>ISTAT – Istituto Nazionale di Statistica (2017a). Classe di addetti e settori economici delle unità locali, anno 2017. Dati scaricati da: http://dati.istat.it/Index.aspx?DataSetCode=DICA_ASIAULP<br>ISTAT – Istituto Nazionale di Statistica (2017b). Censimento delle istituzioni pubbliche, anno 2017. Dati scaricati da: http://dati-censimentipermanenti.istat.it/
	Data of the Park Plan initiatives in 2017	Personal communication from the Circeo National Park
	Data used for the land cover map	ISPRA – Istituto Superiore per la Protezione e la Ricerca Ambientale (2018). Corine Land Cover 2018. Dati scaricati da: http://groupware.sinanet.isprambiente.it/uso-copertura-e-consumo-di-suolo/library/copertura-del-suolo/corine-land-cover/corine-land-cover-2018-iv-livello
**Scientific literature about the case study**	Spatial connectivity and boundary patterns in coastal dune vegetation in the Circeo National Park, Central Italy	Acosta, A., Blasi, C., Stanisci, A. (2000). Spatial connectivity and boundary patterns in coastal dune vegetation in the Circeo National Park, Central Italy. *Journal of Vegetation Science*, 11(1), 149–154. https://doi.org/10.2307/3236787
	Coastal dynamics vs beach users attitudes and perceptions to enhance environmental conservation and management effectiveness	Aretano, R., Parlagreco, L., Semeraro, T., Zurlini, G., Petrosillo, I. (2017). Coastal dynamics vs beach users attitudes and perceptions to enhance environmental conservation and management effectiveness. *Marine Pollution Bulletin*, 123(1–2), 142–155. https://doi.org/10.1016/j.marpolbul.2017.09.003
	Local Community Participation in Italian National Parks Management: Theory versus Practice	Buono, F., Pediaditi, K., Carsjens, G. J. (2012). Local Community Participation in Italian National Parks Management: Theory versus Practice. *Journal of Environmental Policy and Planning*, 14(2), 189–208. https://doi.org/10.1080/1523908X.2012.683937
	Study of seawater intrusion in the coastal areas of Circeo National Park and Litorale Romano Natural Reserve, for the implementation of numerical modelling methods	Manca, F. (2014). *Study of seawater intrusion in the coastal areas of Circeo National Park and Litorale Romano Natural Reserve, for the implementation of numerical modeling methods*. Università Degli Studi Roma Tre, Thesis volume. Pdf file retrieved from: http://hdl.handle.net/2307/4368
	Environmental effects of over-exploitation of the aquifers of the Pontine Plain (Lazio)	Sappa, G., Rossi, M., Coviello, M. (2005). *Effetti ambientali del sovrasfruttamento degli acquiferi della Pianura Pontina (Lazio)*. Aquifer Vulnerability and Risk 2nd International Workshop, 1–16. Pdf file retrieved from: https://www.researchgate.net/publication/260257063_Effetti_ambientali_del_sovrasfruttamento_degli_acquiferi_della_Pianura_Pontina_Lazio

APPENDIX IV – IDENTIFICATION OF ES IN THE CASE STUDY AREA BASED ON THE POLICY DOCUMENTS, according to the Common International Classification of Ecosystem Services (Haines-Young & Potschin, 2011) and the Economics of Ecosystems and Biodiversity (TEEB, 2010) classifications

CNP policy documents		CICES and TEEB classification		
**Datasheet of Environmental Strategic Assessment**	**General and specific objectives and other contents of the Park Plan**	**Examples and indicative benefits**	**Ecosystem service**	**Group**
E8B: Sustainable agricultural supply chain with greenhouse and field cultivation<br>E8C: Promotion and enhancement of sustainable multifunctional agriculture<br>E8D: Realization of a quality/biologic rural-tourist district	G. Ob. 11: Local economic sectors<br>S. Ob. 4.B: Sustainable agriculture<br>Art. 39: Agricultural surfaces	Commercial cropping (cereals, vegetables, fruits)	Food	PROVISIONING
B2D.1: Identification of a scientific management model of the fish populations present in the coastal lakes<br>B3D and B3E: Coordination of socio-economic activities on Paola Lake	S. Ob. 4.C: Fishing and aquaculture<br>Art. 40: Lake surfaces aimed at aquaculture	Commercial fishing (mussels)	Food	
B1H: Sustainable mushroom harvest in the forest	-	Harvesting wild plants and animals for food (mushrooms)	Food	
B1E: Increased suitability of the buildings surrounding the forest for the bats' shelter<br>B1F: Reduction of the load of wild boar in the forest<br>B1G: Elimination of fallow deer disturbance in the forest<br>B2C.2: Creation of suitable habitats for the reproduction of species of waterfowl target in the coastal lakes<br>B4A.5: Observatory implementation for monitoring the migration of birds of prey<br>B5A: Monitoring of Zannone biodiversity<br>B2D.3-4: Numerical control of alien Nutria and tortoises<br>B5B: Management of the mouflon in Zannone<br>C1, C2, C3: Conservation of species and habitats of national, community and international interest	G. Ob. 1-5: Conservation of biodiversity, habitat and species<br>S. Ob. 2: Conservation of species and habitats of national, community and international interest<br>Art. 20: Protection of biodiversity	Maintaining nursery populations (habitat refugees) and maintenance of biodiversity	Lifecycle maintenance and genetic pool protection	REGULATING AND HABITAT

CNP policy documents		CICES and TEEB classification		
**Datasheet of Environmental Strategic Assessment**	**General and specific objectives and other contents of the Park Plan**	**Examples and indicative benefits**	**Ecosystem service**	**Group**
B7B: Sustainable management of the dune system<br>E5: Sustainable development of dune areas	G. Ob. 7: Coastal erosion<br>S. Ob.4.A: Conservation of the dune system<br>S. Ob. 4.A: Sustainable beach management and defence of the dunes<br>Art. 18: Protection of the soil resource	Erosion protection	Erosion prevention	REGULATING AND HABITAT
-	Art. 19: Protection of the quality of the art and the urban environment	Air purification and oxygenation	Air purification	
B2A.1: Improvement of the chemical-physical water quality parameters in coastal lakes<br>B2A.2: Activation of new scoops of Fogliano Lake<br>B2A.3: Activation of agreement protocol with ARPA Lazio on water quality monitoring<br>B3A: Purification of the waters of Paola Lake<br>E11: Sustainable management of the water and material cycle	Art. 17: Protection of water resources	Water purification and oxygenation	Water purification	
B1A: Sustainable use of the forest<br>B2C.1: Regulation and organization of tourist access to the shores of coastal lakes<br>B2C.3: Construction of a Monitoring Centre for the coastal wetlands of the Park<br>B4A.2: Sport climbing regulation<br>B4A.3: Tourist use of the Promontory<br>B4C.1: Recovery and use of the promontory caves<br>B5C: Sustainable development of the island of Zannone<br>B5F: Hospitable Zannone<br>B7B: Sustainable management of the dune system<br>E5: Sustainable development in dune areas<br>E6A: Sustainable tourism development plan for the Island of Zannone	G. Ob. 11: Local economic sectors<br>S. Ob. 4.A: Naturalistic tourism<br>S. Ob. 4.A: Sustainable beach management and defence of the dunes<br>Art. 35: Areas of environmental interest compatible with touristic and hotel activities<br>Map: Ecotourism	Recreational and sports activities	Recreation and tourism	CULTURAL
B4B.4: Hydrogeological risk management in a way that is compatible with the environmental and landscape value	G. Ob. 12: Landscape, archaeological, monumental, historical and cultural heritage<br>Art. 24: Protection and enhancement of heritage of historical, architectural and environmental value<br>Art. 27: Areas of particular historical and landscape value<br>Map: Landscape heritage	Areas of outstanding natural beauty and tranquillity	Aesthetic values	
F1: Plan of the researches and of the monitors considered priority<br>F2: Information and environmental education	S. Ob. 5: Promotion and promotion of research, monitoring, information and environmental education activities	Scientific research and environmental education	Information for cognitive development	

APPENDIX V – DISTRIBUTION (IN %) OF QUESTIONNAIRE RESPONDENTS, MAP OF HOUSE AND WORK AREAS IN THE QUESTIONNAIRE

Gender	
F	48
M	52
**Age**	
18-24	7.7
25-34	15.2
35-44	18.4
45-54	19.1
55-64	14.9
65-74	13.2
>75	11.6
**Qualification**	
Elementary school	4.9
High school	46.4
Master	3.7
Middle school	15.3
None	0.6
PhD	0.5
University	28.6
**Occupation**	
No answer	2.6
Disabled and/or unsuitable	0.9
Full time worker	39.7
Housework or assistance	4.6
Inactive	0.1
Part time worker	9
Retired or Cessation	27.7
Student/Professional training	8.3
Unemployed	6.3
Unpaid work experience	0.7
**Business sectors**	
Agriculture. Livestock and Forestry	9.8
Fishing and Aquaculture	1.5
Industry and Crafts (transformation of raw materials)	8.7
Buildings	1.1
Wholesale and retail trade	6.4
Hotel and catering sector	6.3
Financial. insurance and real estate activities	3.3
Education	11.9
Public administration and defence	16.1
Professional. scientific. and technical activities	9.8
Rental. travel agencies. business support services	1.4
Transport and storage	0.2
Other activities	23.6

Municipality of residence	
Sabaudia	66.7
San Felice Circeo	33.3
**House Area**	
A (CNP area)	1.5
B	11.2
C (CNP area)	40.8
D	6.7
E (CNP area)	7.8
F	19.6
G (CNP area)	12.3
**Work area**	
A (CNP area)	0.8
B	3.7
C (CNP area)	18.1
D	2
E (CNP area)	4.5
F	8.2
G (CNP area)	6.9
No work	35.1
Other areas	20.6
**Proximity to the Park**	
House	62.4
Work	30.3
House & Work	24.4

0

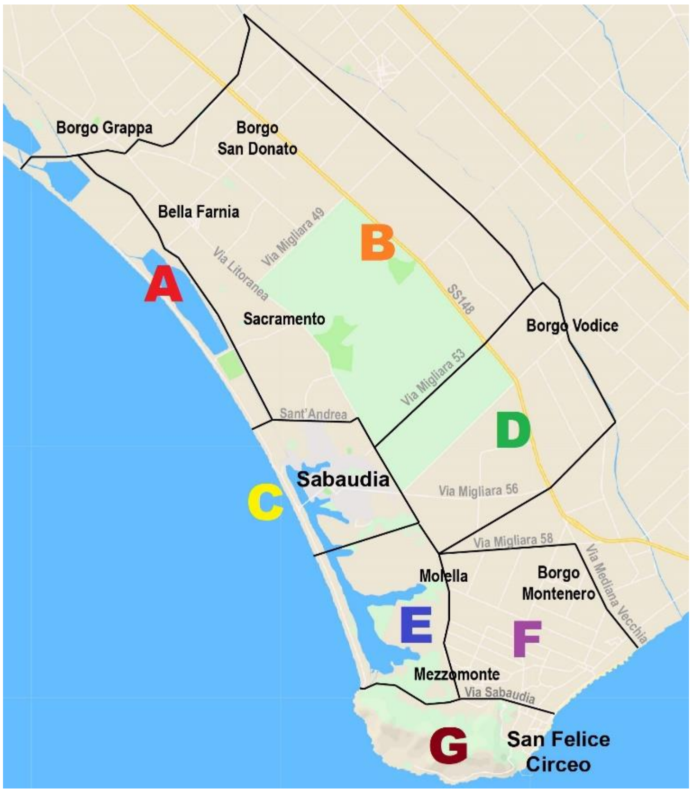

APPENDIX VI – QUESTIONNAIRE FOR THE CASE STUDY (UNDERLINED QUESTIONS HAVE BEEN USED FOR THE RESULTS OF THIS PUBLICATION)

PERCEPTION OF CIRCEO NATIONAL PARK

Welcome to the "Perception of Circeo National Park" questionnaire. The survey, carried out as part of a Ph.D. research at La Sapienza University, aims to analyse the perception and importance of Circeo National Park for the citizens of Sabaudia and San Felice Circeo. The questionnaire is anonymous, and the data collected will be treated in an aggregate manner, in compliance with the law on privacy. This analysis is carried out in collaboration with Circeo National Park and, therefore, the results can be used by the Park in order to improve its services.

*** Questions marked with asterisk are mandatory**

Perception of Circeo National Park territory

The first sections of the questionnaire will ask to answer the questions based on your knowledge and personal opinions. Feel free to answer in total sincerity as the questionnaire is anonymous and the data collected will be treated in an aggregate manner, in compliance with the law on privacy.

1. **Which of the following natural environments are part of Circeo National Park?**
   You can mark one or more answers

   ☐ Forest

   ☐ Circeo Promontory

   ☐ Paola and Caprolace Lakes

   ☐ Fogliano and Monaci Lakes

   ☐ Sabaudia dunes

   ☐ San Felice Circeo dunes

   ☐ Islands of Ponza and Palmarola

   ☐ Island of Zannone

   ☐ None

2. **Which of the following inhabited areas are part of Circeo National Park?**
   You can mark one or more answers

   ☐ Sabaudia city

   ☐ Old town of San Felice Circeo

   ☐ Molella

   ☐ Baia d'Argento

   ☐ None

3. **On which of the following Municipalities does Circeo National Park territory extend?**
   You can mark one or more answers

   ☐ Latina

   ☐ Ponza

   ☐ Sabaudia

   ☐ San Felice Circeo

Perception of Circeo National Park activities

4. Which of the following activities are carried out by Circeo National Park?
You can mark one or more answers

☐ Firefighting

☐ Forest Rangers

☐ Authorizations

☐ Summer events organization

☐ Agriculture promotion

☐ Touristic promotion

☐ Excursions and environmental education

☐ None

5. Who manages Circeo National Park?
You can mark one or more answers

☐ Sabaudia Municipality

☐ San Felice Circeo Municipality

☐ Forest Rangers

☐ Park Authority

☐ Lazio Region

6. How does the presence of Circeo National Park impact on its activities or those of its family? *
You can mark only one answer

◯ In no way

◯ Negatively

◯ Positively

7. Why?

____

____

____

Trust and Communication

8. **What is your level of confidence in the management of Circeo National Park and in the resolution of problems and tensions with the citizens?**
   You can mark only one answer

	1	2	3	4	
Very low	◯	◯	◯	◯	Very high

9. **Select the themes that are taken into consideration by Circeo National Park in the implementation of initiatives and/or events.**
   You can mark one or more answers

☐ Agriculture

☐ Cultural heritage

☐ Biodiversity

☐ Deterioration and environmental pollution

☐ Park presentation

☐ Promotion of the territory

☐ Tourism

☐ None

10. **How do you assess the communication of the initiatives organized by Circeo National Park?**
    You can mark only one answer per row

	I don't know	Inexistent	Low	Sufficient	High
Local press	◯	◯	◯	◯	◯
Social Networks	◯	◯	◯	◯	◯
Newsletter	◯	◯	◯	◯	◯
Word of mouth	◯	◯	◯	◯	◯
Posters	◯	◯	◯	◯	◯

Participation

11. Which of the following Circeo National Park initiatives did you participate? *
You can mark one or more answers

☐ Training activities

☐ Park Plan meetings

☐ Meetings with farmers

☐ Meetings with touristic operators

☐ Promotional meetings

☐ Cultural heritage meetings

☐ Biodiversity meetings

☐ Deterioration and environmental pollution meetings

☐ None

12. If you did not participate in any initiative, why?
You can mark only one answer

○ I was aware of it, but I'm not interested in this kind of initiative

○ I was aware of it, but I never got a chance/time/opportunity to participate

○ I was not aware of it, but I would not have participated anyway

○ I was not aware of it, but I would have considered whether to participate

○ Other: ______________________

13. Which of the following Circeo National Park touristic events did you participate? *
You can mark one or more answers

☐ Environmental education

☐ Food and wine

☐ Cultural conferences

☐ Summer initiatives

☐ Sport initiatives

☐ Guided tours

☐ None

14. If you did not participate in any initiative, why?
You can mark only one answer

○ I was aware of it, but I'm not interested in this kind of initiative

○ I was aware of it, but I never got a chance/time/opportunity to participate

○ I was not aware of it, but I would not have participated anyway

○ I was not aware of it, but I would have considered whether to participate

○ Other: ______________________

Future perspectives

15. In order to improve the management of Circeo National Park and communication with citizens, are you available to participate in the following initiatives? *

You can mark only one answer per row

	Not available	Hardly available	Quite available	Very available
Meetings with inhabitants of Circeo National Park	◯	◯	◯	◯
Meetings with workers	◯	◯	◯	◯
Training meetings	◯	◯	◯	◯
Focus groups to solve conflicts	◯	◯	◯	◯
Administrative meetings	◯	◯	◯	◯

16. In order to improve the management of Circeo National Park and communication with citizens, are you available to use the following services? *

You can mark only one answer per row

	Not available	Hardly available	Quite available	Very available
Information desk at the visitor centre	◯	◯	◯	◯
Discussion forum on the website Parcocirceo.it	◯	◯	◯	◯
Online survey of citizens' satisfaction	◯	◯	◯	◯
Social networks as information moment	◯	◯	◯	◯

17. Other suggestions?

____

____

____

Perception of Circeo National Park benefits

This section wants to evaluate which benefits of Circeo National Park are important for the well-being of society and citizens. Therefore, it will ask to assess the importance of some benefits for social well-being and then for personal well-being.

18. Do you consider the following statements are true? *
You can mark only one answer per row

	Yes	No	I don't know
Agricultural goods are cultivated in some areas of Circeo National Park	◯	◯	◯
Different fishes and mussels are caught in Paola Lake	◯	◯	◯
Picking mushrooms is possible in some areas of Circeo National Park with a special license	◯	◯	◯
Circeo National Park vital spaces, refuges and protection areas for various animal species	◯	◯	◯
Vegetation of Circeo National Park has an important role in the soil erosion control	◯	◯	◯
Environments of Circeo National Park has an important role in the air purification	◯	◯	◯
Environments of Circeo National Park has an important role in the water purification	◯	◯	◯
Recreation, touristic and sport activities are possible in different areas Circeo National Park	◯	◯	◯
Some areas of Circeo National Park have landscape values and provide benefits for human health	◯	◯	◯
Some areas of Circeo National Park have educational and scientific value	◯	◯	◯

19. Consider following groups of benefits provided by Circeo National Park. Which group is the most important for the SOCIAL well-being? *
You can mark only one answer

	Description	Examples
Provisioning	Benefits obtained from the consumption of resources and raw materials produced by nature	Oxygen<br>Water<br>Food
Regulating	Benefits related to natural functions that allow the maintenance of human health and the functioning of the environments	Climate regulation<br>Water depuration<br>Soil erosion control<br>Habitat for species
Cultural	Benefits population gets from nature, through spiritual enrichment, cognitive development, recreational and aesthetic experiences	Recreation activities<br>Educational values<br>Wellness of being in nature<br>Sense of identity<br>Aesthetic value

◯ Provisioning
◯ Regulating
◯ Socio-cultural

20. And which group is the second most important for the SOCIAL well-being? *
You can mark only one answer per row

◯ Provisioning
◯ Regulating
◯ Socio-cultural

21. **<u>Consider the following list of benefits provided by Circeo National Park. Evaluate each of them based on what you consider important for your PERSONAL well-being</u>** *

You can mark only one answer per row

	Photo	Description	Examples
Food from agriculture	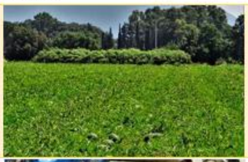	Crops for human consumption from agricultural areas of CNP (Molella, Palazzo, Sant'Andrea, etc.)	Turnips, Carrots, Watermelons, Zucchini
Food from aquaculture	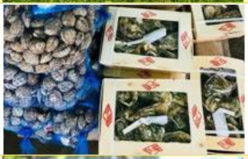	Fish for human consumption from Paola Lake	Mussels, clams, mullets
Food from mushrooms picking	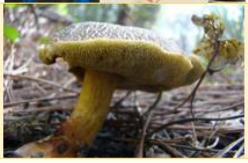	Wild edible mushrooms in the areas of CNP	Different types of mushrooms

	Not important	Not very important	Quite important	Very important
Food from agriculture				
Food from aquaculture				
Food from mushrooms picking				

22. **<u>Consider the following list of benefits provided by Circeo National Park. Evaluate each of them based on what you consider important for your PERSONAL well-being</u>** *

You can mark only one answer per row

	Photos	Description	Examples
Habitat for species	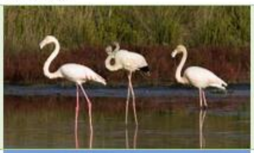	Provision of suitable living and nursing places for wild species	Birds, Wild boards, Fallow deers
Soil erosion control	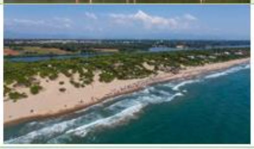	Role of vegetation root matrix and soil biota in soil retention	Retention of soil via plants roots
Air purification	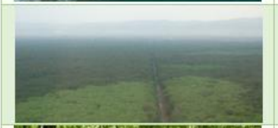	Role of natural ecosystems in the air decontamination	Clean air
Water purification	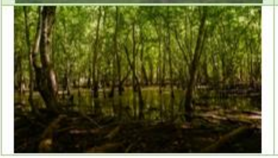	Filtering, retention and storage of fresh water	Clean water for consumptive use (drinking, irrigation and industrial use)

	Not important	Not very important	Quite important	Very important
Habitat for species				
Soil erosion control				
Air purification				
Water purification				

23. Consider the following list of benefits provided by Circeo National Park. Evaluate each of them based on what you consider important for your PERSONAL well-being *

You can mark only one answer per row

	Photos	Description	Examples
Nature recreation activities	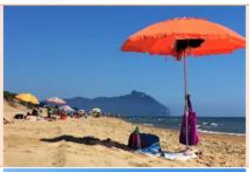	Possibility to benefits by outdoor, touristic, and sportive activities by natural ecosystem	Hiking, Cycling, Bathing, Birdwatching
Aesthetic value and tranquillity of nature	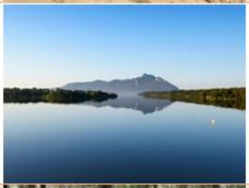	Attractive landscape features and peaceful landscape for the human physical and psychological well-being	Pleasure of beautiful views<br>Pleasure of being in nature
Environmental education and science	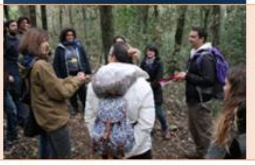	Ecosystem features of educational and scientific value	School visits and Research

	Not important	Not very important	Quite important	Very important
Nature recreation activities	◯	◯	◯	◯
Aesthetic value and tranquillity of nature	◯	◯	◯	◯
Environmental education and science	◯	◯	◯	◯

Personal information

This section will ask some personal information for purely statistical purposes. Remember that the questionnaire is anonymous, and the data collected will be treated in an aggregate manner, in compliance with the law on privacy.

24. Gender

You can mark only one answer

- M
- F
- Rather not answer

25. Age

You can mark only one answer

- 18-24
- 25-34
- 35-44
- 45-54
- 55-64
- 65-74
- 75 and more

26. Nationality

You can mark only one answer

- Italian
- Other: ______

27. Qualification

You can mark only one answer

- Elementary school
- Middle school
- High school
- University
- Master
- Ph.D.
- None

28. Occupation

You can mark only one answer

- Full time worker
- Part time worker
- Unemployed
- Inactive
- Student/Professional training
- Unpaid work experience
- Retired or Cessation
- Disabled and/or unsuitable
- Housework or assistance
- Other: ______

29. <u>Business sector</u>
You can mark one or more answers

☐ Agriculture, Livestock, and Forestry

☐ Fishing and Aquaculture

☐ Industry and Crafts (transformation of raw materials)

☐ Buildings

☐ Wholesale and retail trade

☐ Hotel and catering sector

☐ Rental, travel agencies, business support services

☐ Transport and storage

☐ Financial, insurance and real estate activities

☐ Professional, scientific and technical activities

☐ Education

☐ Public administration and defence

☐ Other activities

30. Residence *
You can mark only one answer

◯ Sabaudia

◯ San Felice Circeo

31. How long have you lived here?
You can mark only one answer

◯ Always

◯ Less than 5 years

◯ More than 5 years

32. **<u>Consider the areas delimited by black lines and marked with coloured letters on the map. In which area is his house located?</u>** *

You can mark only one answer

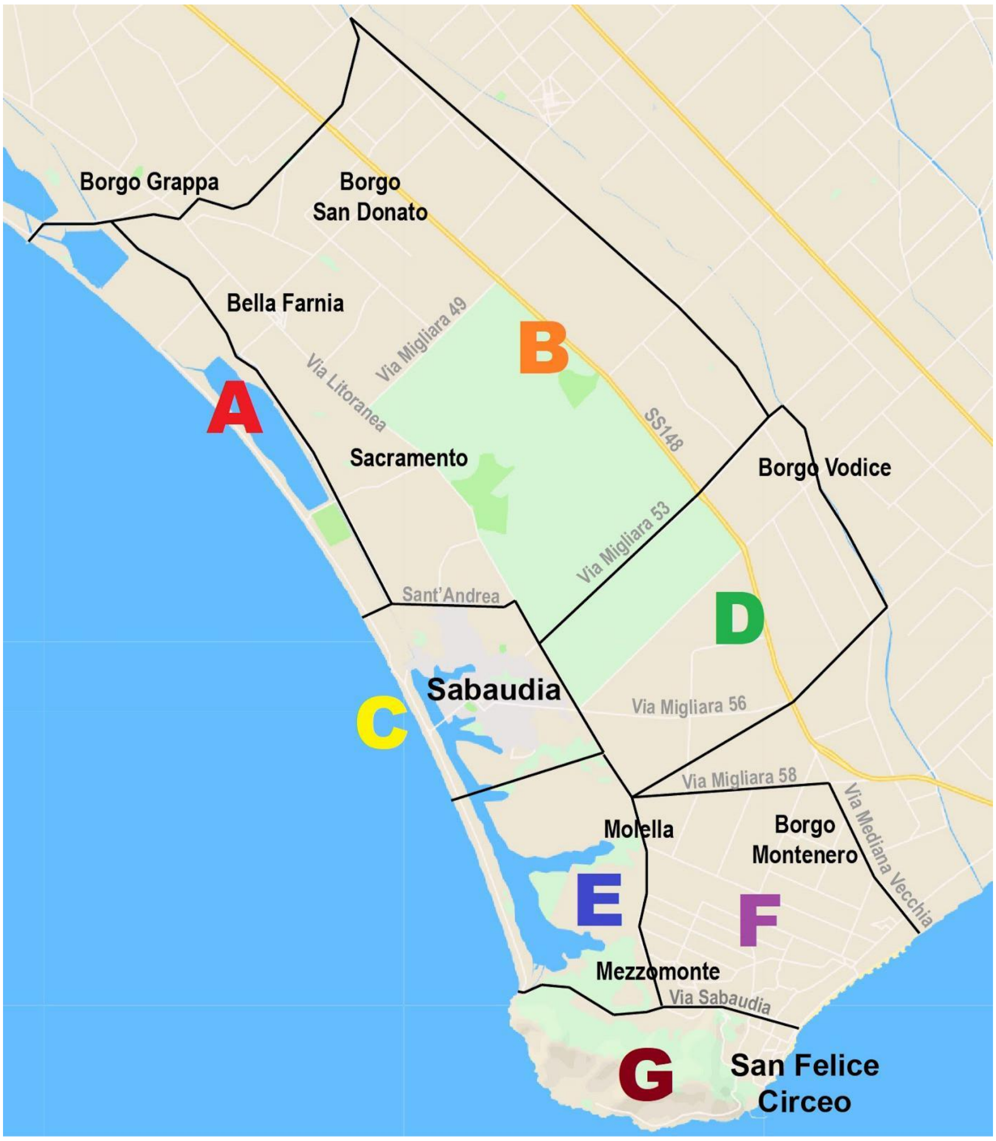

- Area with letter A
- Area with letter B
- Area with letter C
- Area with letter D
- Area with letter E
- Area with letter F
- Area with letter G

33. **Consider the same areas as the previous map. In which area is your main working activity?** *

You can mark only one answer

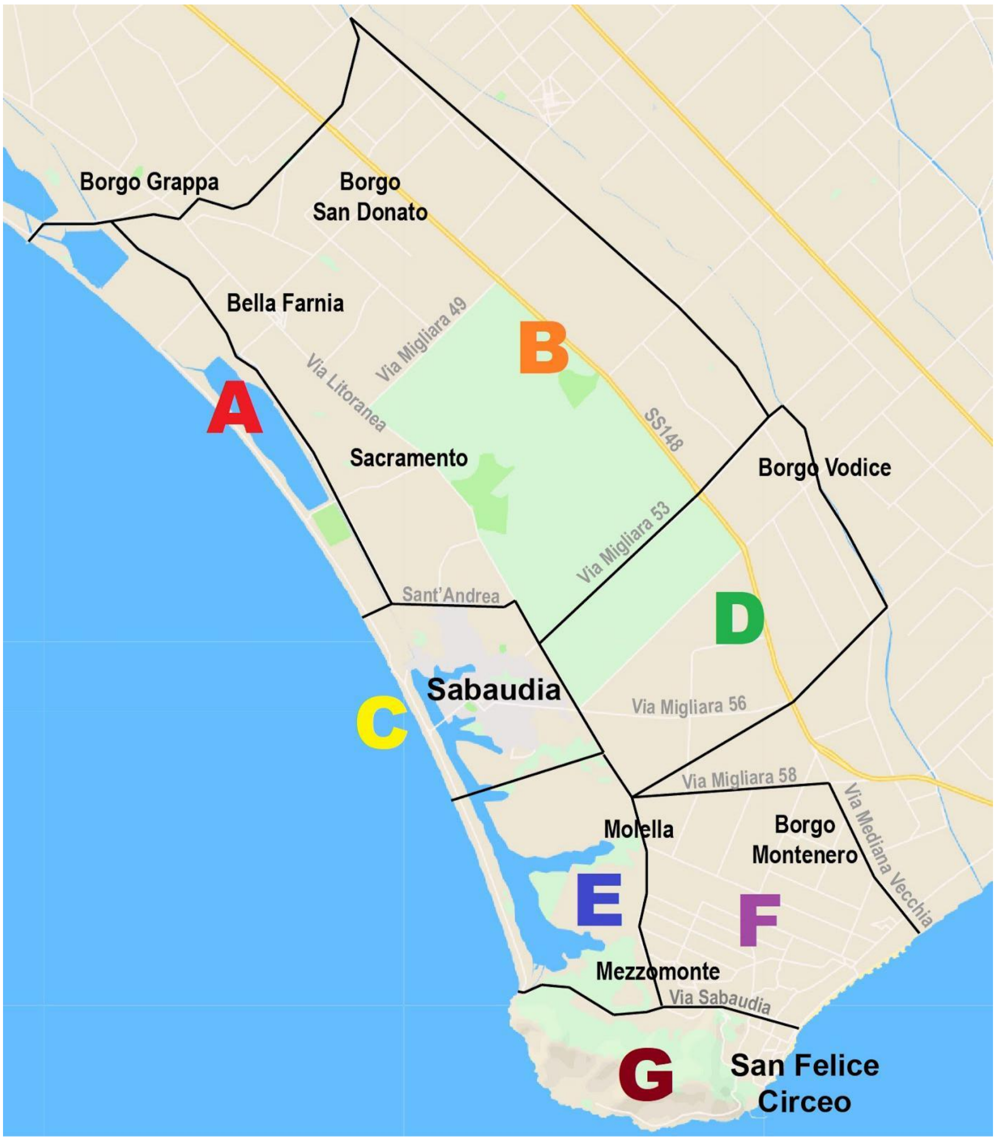

- Area with letter A
- Area with letter B
- Area with letter C
- Area with letter D
- Area with letter E
- Area with letter F
- Area with letter G
- In none of the areas marked with letters
- I don't have a job

THANKS FOR YOUR PARTICIPATION!

APPENDIX VII – CIRCEO NATIONAL PARK ZONING MAP (ENTE PARCO NAZIONALE DEL CIRCEO, 2011e)

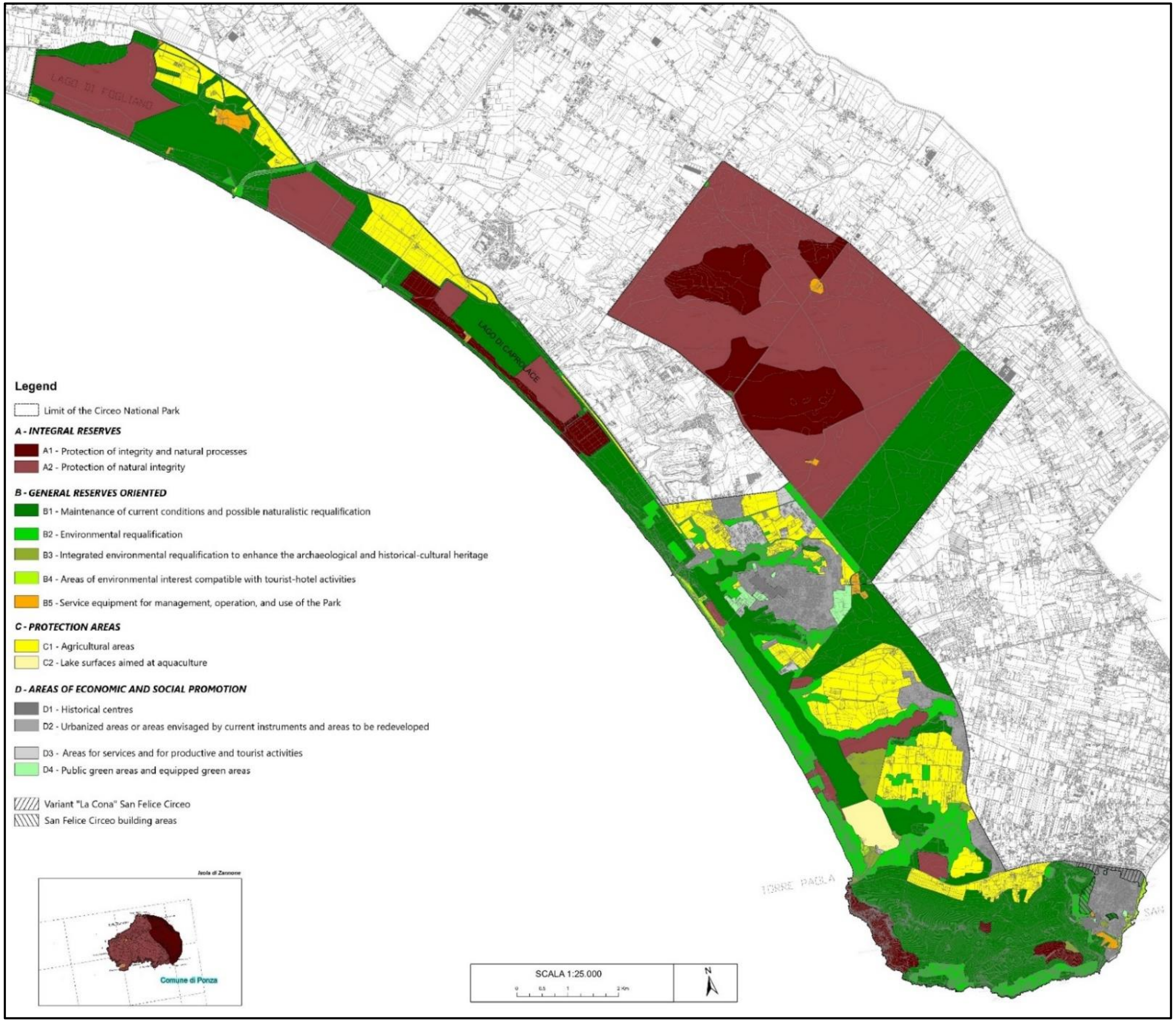

Supplement: Appendices [file TBSM_A_1946155_SM3139.pdf]
